# Ketogenic diet and ketone bodies enhance the anticancer effects of PD-1 blockade

**DOI:** 10.1172/jci.insight.145207

**Published:** 2021-01-25

**Authors:** Gladys Ferrere, Maryam Tidjani Alou, Peng Liu, Anne-Gaëlle Goubet, Marine Fidelle, Oliver Kepp, Sylvère Durand, Valerio Iebba, Aurélie Fluckiger, Romain Daillère, Cassandra Thelemaque, Claudia Grajeda-Iglesias, Carolina Alves Costa Silva, Fanny Aprahamian, Déborah Lefevre, Liwei Zhao, Bernhard Ryffel, Emeline Colomba, Monica Arnedos, Damien Drubay, Conrad Rauber, Didier Raoult, Francesco Asnicar, Tim Spector, Nicola Segata, Lisa Derosa, Guido Kroemer, Laurence Zitvogel

**Affiliations:** 1 Gustave Roussy, INSERM U1015, Villejuif, France.; 2 Equipe labellisée par la Ligue contre le cancer, Université de Paris, Sorbonne Université, INSERM UMR1138, Centre de Recherche des Cordeliers, Paris, France.; 3 Department of Medical Sciences, University of Trieste, Trieste, Italy.; 4 EverImmune, Villejuif, France.; 5 INEM — UMR7355, CNRS and Université Orléans, France.; 6 Gustave Roussy Hospital, Villejuif, France.; 7 Gustave Roussy, Department of Biostatistics and Epidemiology, University Paris-Saclay, Villejuif, France.; 8 URMITE, Aix Marseille Université, UM63, CNRS 7278, IRD 198, INSERM 1095, IHU-Méditerranée Infection, Marseille, France.; 9 Department Cellular, computational and integrative biology (CIBIO), University of Trento, Trento, Italy.; 10 The Department of Twin Research & Genetic Epidemiology, King’s College London, United Kingdom.; 11 University Paris Saclay, Saint-Aubin, France.; 12 CIC 1428 BIOTHERIS, Gustave Roussy, Villejuif, France.

**Keywords:** Metabolism, Oncology, Cancer, Immunotherapy, Mouse models

## Abstract

Limited experimental evidence bridges nutrition and cancer immunosurveillance. Here, we show that ketogenic diet (KD) — or its principal ketone body, 3-hydroxybutyrate (3HB), most specifically in intermittent scheduling — induced T cell–dependent tumor growth retardation of aggressive tumor models. In conditions in which anti–PD-1 alone or in combination with anti–CTLA-4 failed to reduce tumor growth in mice receiving a standard diet, KD, or oral supplementation of 3HB reestablished therapeutic responses. Supplementation of KD with sucrose (which breaks ketogenesis, abolishing 3HB production) or with a pharmacological antagonist of the 3HB receptor GPR109A abolished the antitumor effects. Mechanistically, 3HB prevented the immune checkpoint blockade–linked upregulation of PD-L1 on myeloid cells, while favoring the expansion of CXCR3^+^ T cells. KD induced compositional changes of the gut microbiota, with distinct species such as *Eisenbergiella massiliensis* commonly emerging in mice and humans subjected to carbohydrate-low diet interventions and highly correlating with serum concentrations of 3HB. Altogether, these results demonstrate that KD induces a 3HB-mediated antineoplastic effect that relies on T cell–mediated cancer immunosurveillance.

## Graphical abstract

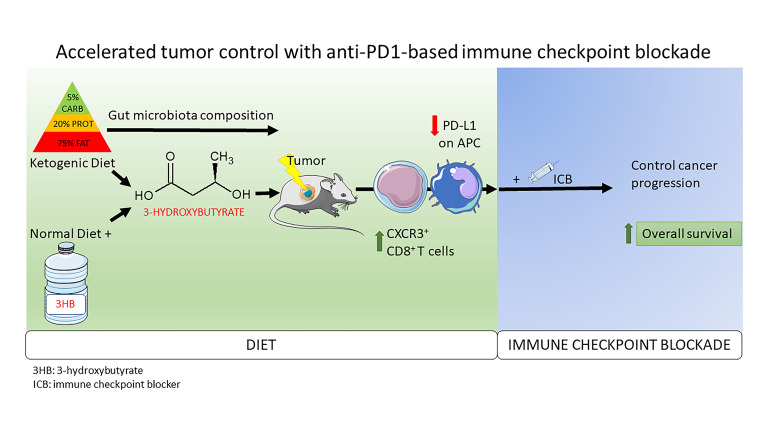

## Introduction

The clinical optimization of immune checkpoint inhibitors has provoked a paradigm shift in the treatment of advanced cancers, implementing immunotherapy as a first- and second-line modality across many tumor types (1). The major advance of immunotherapy concerns the long-term increase in overall survival, as observed for treatment with antibodies targeting cytotoxic T-lymphocyte-associated protein 4 (CTLA-4), programmed cell death 1 (PD-1), or programmed death–ligand 1 (PD-L1), compared with other standard therapies such as chemotherapy or tyrosine kinase inhibitors (2). However, 2 limitations affect this medical breakthrough. First, only a subset of cancers within each indication (melanoma, lung, kidney, bladder, head and neck tumors, etc.) responds to immune checkpoint blockade (ICB), leaving the majority of patients without clinical benefit. Second, immune-related adverse events are frequent, largely unpredictable, and often debilitating, limiting the clinical use of ICB (3). Several factors, including host genetics, the tumor mutational load, as well as environmental cues, may determine immune responses against cancer (4). While most efforts to understand cancer immune resistance largely focused on somatic alterations of cancer cells and their effects on the tumor microenvironment and lymphoid organs, relatively little information is available about environmental factors affecting the delicate balance between metabolism, microbiota, immunity, and oncogenesis (5–8). Potentially relevant environmental factors encompass diet, lifestyle (such as stress, exercise, and circadian rhythm), and past or present exposures to infectious agents and xenobiotics. Among these, the most easily actionable factor is diet. Food not only represents a source of macronutrients and micronutrients required for fueling metabolism, but it also affects the homeostatic maintenance of intestinal barrier integrity, the composition of microbiota, and the function of the immune system. To date, a plethora of dietary patterns and functional supplements (nutraceuticals) have been assessed for potential health benefits, including cancer prevention (9). In 2010, the United Nations Educational, Scientific and Cultural Organization (UNESCO) recognized the Mediterranean diet as an “intangible cultural heritage of humanity” (10). Calorie restriction reduces the risk of chronic disease, including cancer, and increases life expectancy by promoting autophagy (11, 12). Aside from caloric restriction, low protein consumption may restrain inflammation and improve anticancer immunosurveillance (13, 14). Recently, several studies have suggested positive therapeutic effects of very low–carbohydrate ketogenic diets (KD) on several diseases, including cancer (15–17).

First described by Hans Krebs, ketosis is a metabolic state in which the body retrieves energy from the catabolism of ketone bodies (KB), as opposed to the normal state when glucose — and, to a lesser degree, fatty acids and amino acids — is the main source of energy. KB include acetylacetate (H3C–CO–CH2–COO–), β–D-hydroxybutyrate (H3C–CHOH–CH2–COO–), and volatile acetone (H3C–CO–CH3). KD are high-fat, moderate-protein, and low-carbohydrate (usually less than 40 g/day) diets (18), and they induce a surge in KB — in particular, 3-hydroxybutyrate (3HB). This increase in 3HB also occurs after fasting, favoring mitochondrial respiration rather than glycolysis for energy metabolism. So far, the rationale to use KD to combat cancer was based on a cell-autonomous hypothesis. One hallmark of all cancer cells is the abnormal metabolic phenotype first described by Otto Warburg, which is characterized by a bioenergetic shift from oxidative phosphorylation toward glycolysis (19, 20). The Warburg phenomenon is linked to the overactivation of the insulin/IGF-1–dependent phosphatidylinositol 3-kinase (PI3K)/AKT/mTOR system, not only due to mutations in the genes that code for pathway proteins, but also due to chronic hyperglycemia and hyperinsulinemia (21–24). Therefore, a potential therapeutic strategy might involve KD, which reduces glucose availability to tumor cells, while providing KB as an alternative bioenergetic fuel to normal cells. On theoretical grounds, this should result in selective starvation of tumor cells, which are unable to adapt to ketone metabolism as a result of their acquired metabolic inflexibility and genomic instability. This is emphasized by the fact that various tumors display reduced levels of 2 enzymes involved in KB catabolism (O-hydroxy-butyrate dehydrogenase and succinyl-CoA-acetoacetate-CoA transferase) (25–28).

Several diseases involving alterations in mitochondrial metabolism — such as epilepsy (29), diabetes mellitus type II, obesity, neurological disorders, and cancer — may benefit from KD (30–33). However, most of the knowledge of the beneficial effects of KD on cancer relies on a cell-autonomous vision of malignancy, as well as on epidemiological studies rather than experimental animal models. We and others highlighted the intertwined relationship between the composition of the microbiota and antitumor immune responses in advanced-cancer bearers treated with immunotherapy (8, 34). Therefore, bridging the gap linking dietary habits to clinical outcomes following immunotherapy remains an open challenge.

Here, we use preclinical tumor models to address the functional impact of KD and KB on the host immune system and tumor immunosurveillance, highlighting the immunomodulatory properties of KD and the most abundant KB, which is 3HB. We found both that KD and 3HB influence the balance between immunostimulatory and immunosuppressive pathways, thereby improving the antitumor effects of ICB.

## Results

### KD retards tumor growth in aggressive tumor models.

**Figure 1:**
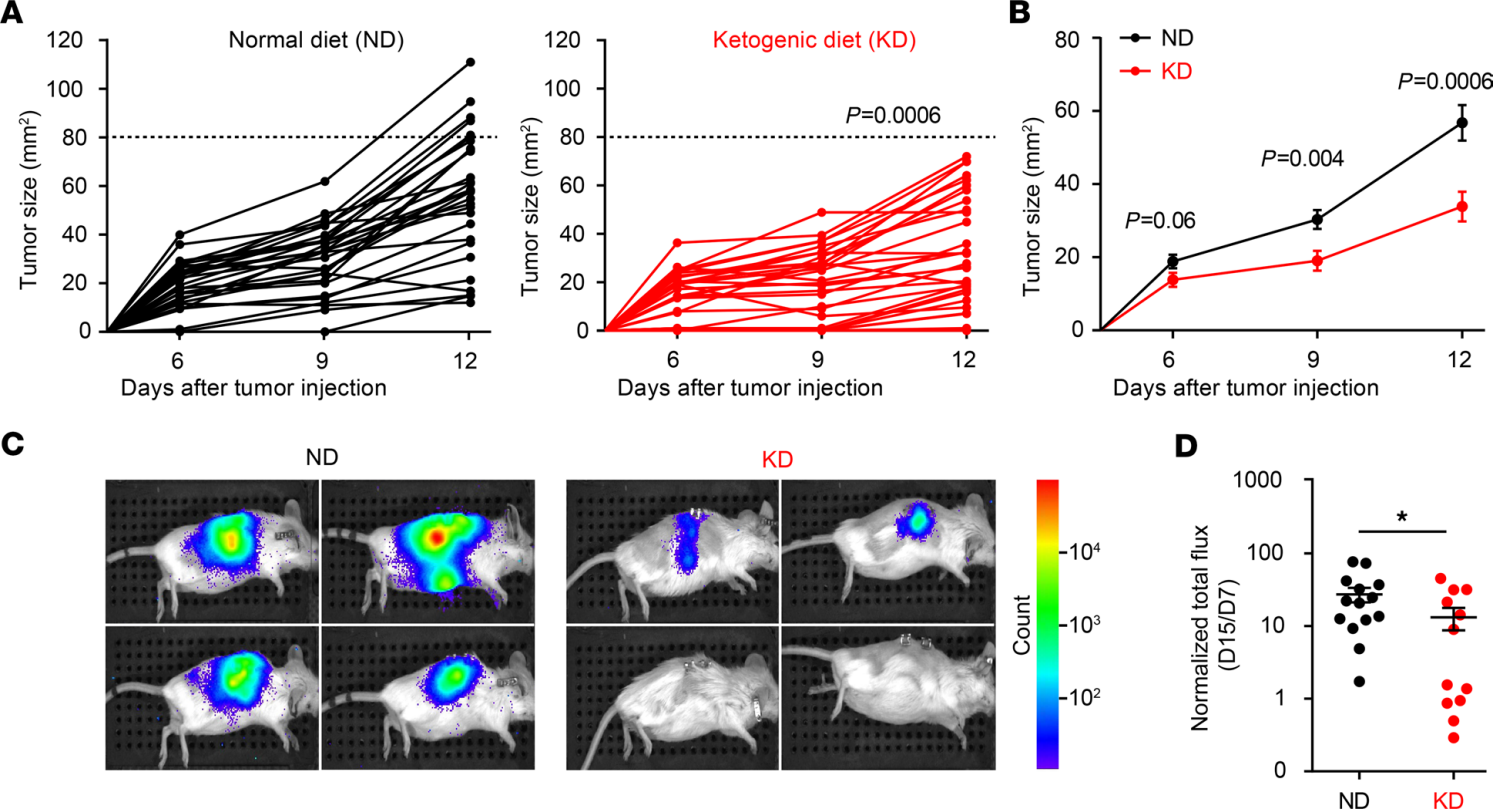
Ketogenic diet decreases melanoma and renal cell tumor growth. (**A–B**) Growth kinetics of RET melanoma in C57BL/6J mice. The minimal tumorigenic dose of RET melanoma cells was inoculated s.c. the day of starting the dietary intervention. Normal diet (ND) versus ketogenic diet (KD). Tumor size was monitored 3 times a week for 12 days. Each curve represents the tumor kinetics of 1 animal (**A**), and the mean ± SEM of tumor size was calculated for both nutritional interventions (**B**). The graphs depict the concatenation of 5 independent experiments involving 5–6 mice/group (*n* = 29–30 mice). (**C**) Monitoring of RENCA progression using bioluminescence imaging of luciferase activity in 4 representative mice among 15 BALB/c mice fed ND versus KD. (**D**) The ratio of luminescence at day 15 (D15) versus day 7 after orthotopic tumor injection was calculated for each diet (D15, IVIS measurement; D7, day of randomization). All experiments were composed of 5–7 mice/group and were performed at least twice, yielding similar results (*n* = 14 for ND and *n* = 12 for KD). Dedicated software (https://kroemerlab.shinyapps.io/TumGrowth/) (**A** and **B**) and Student’s *t* test. *P < 0.05 (**D**).

We analyzed the antitumor effects of an ad libitum nutritional KD (called KetoCal) based on a 4:1 fat/protein ratio compared with a vitamin and mineral-matched control diet (normal diet, ND) in C57BL/6J and BALB/c mice reared in specific pathogen–free (SPF) conditions. KD was initiated the day of tumor inoculation and pursued for 12 consecutive days, and tumor progression was monitored twice a week. As compared with ND, KD reduced the growth of transgenic melanoma (RET) inoculated orthotopically into the skin (Figure 1, A and B). Moreover, KD delayed the progression of a luciferase-expressing renal cancer (RENCA) implanted into the subcapsular area of kidneys from BALB/c mice, preventing tumor outgrowth in less than half of cases, while the other animals harbored smaller tumors (Figure 1, C and D). These effects advantageously compared with regimens of short-term starvation or food supplements that we used in the past to improve anticancer immunosurveillance (12, 35).

Thus far, in accordance with the literature dealing with other cancer models (35–39), KD attenuated tumor progression in orthotopic models of aggressive neoplasia.

### 3HB is the bioactive metabolite of KD.

**Figure 2:**
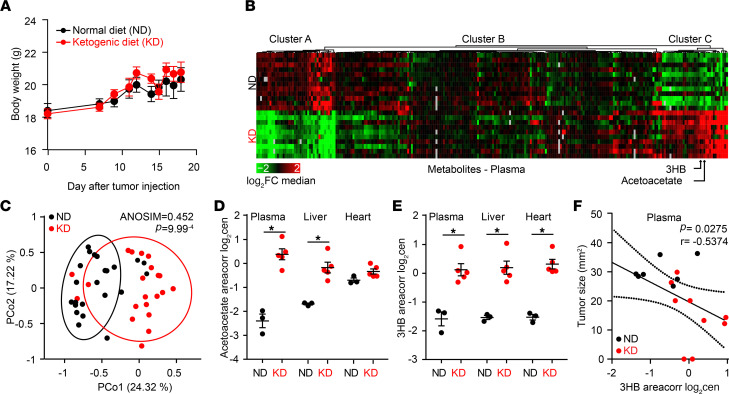
Ketone bodies accumulate in tissues of ketogenic diet fed mice. (**A**) Follow up of mouse weight over time until sacrifice in tumor bearers fed normal diet (ND) versus ketogenic diet (KD) (*n* = 30 for each groups). (**B** and **C**) Heatmap of the nonsupervised hierarchical clustering highlighting differences in the metabolic profiling of C57BL/6J mice fed ND (*n* = 20) versus KD (*n* = 21) in plasma (**B**) and PCA based on Bray-Curtis Dissimilarity Index showing significant compositional differences across diet types (**C**) (PERMANOVA, *P* = 0.001). Arrows indicate 3-hydroxybutyrate (3HB) and acetoacetate positions in the heatmap. (**D** and **E**) Concentrations of acetoacetate and 3HB in 3 compartments of tumor bearers. A representative experiment involving 5 mice/group is depicted out of 2 metabolomics profiles yielding similar results (*n* = 3 in ND and *n* = 5 in KD). (**F**) Spearman correlations between levels of 3HB in plasma and RET tumor size at day 12 indicating *r* and *P* values for the KD group. Not significant for the ND group. ANOVA (**A**) and Student’s *t* test statistical analyses of mean ± SEM. *P < 0.05 (**D** and **E**).

There were no significant differences in food consumption or weight gain between mice fed KD versus ND (Figure 2A). To identify the metabolic changes associated with ad libitum KD in tumor bearers, we performed a comprehensive metabolomic profiling of plasma, liver, and heart tissues from 24 RET melanoma–bearing mice receiving either ND or KD at day 10, with the time corresponding to disease control for most animals. Nonsupervised hierarchical clustering and principal coordinate analyses (PCA) revealed major differences between ND and KD (Figure 2, B and C, and Supplemental Table 1; supplemental material available online with this article; https://doi.org/10.1172/jci.insight.145207DS1). KB including acetoacetate, 2-hydroxybutyrate (2HB), and 3HB — as well as carnitine derivatives (such as 3-methylglutaryl carnitine and the acetyl carnitine/carnitine ratio), short-chain saturated carboxylic acids (such as behenic acid), and polyamines (in particular, N1-acetyl spermidine and the putrescine/ornithine ratio) — were markedly elevated in the plasma and other tissues from mice fed with KD (Figure 2, D and E; Supplemental Table 1; and Supplemental Figure 1). In contrast, acetylcholine, a range of acyl phosphatidylcholines (PCae), palmitate (the most abundant saturated fatty acid), several monounsaturated fatty acids, propionate, and hippurate were significantly decreased by KD compared with ND (Figure 2B and Supplemental Table 1), suggesting the catabolism of fatty acids through β-oxidation.

**Figure 3:**
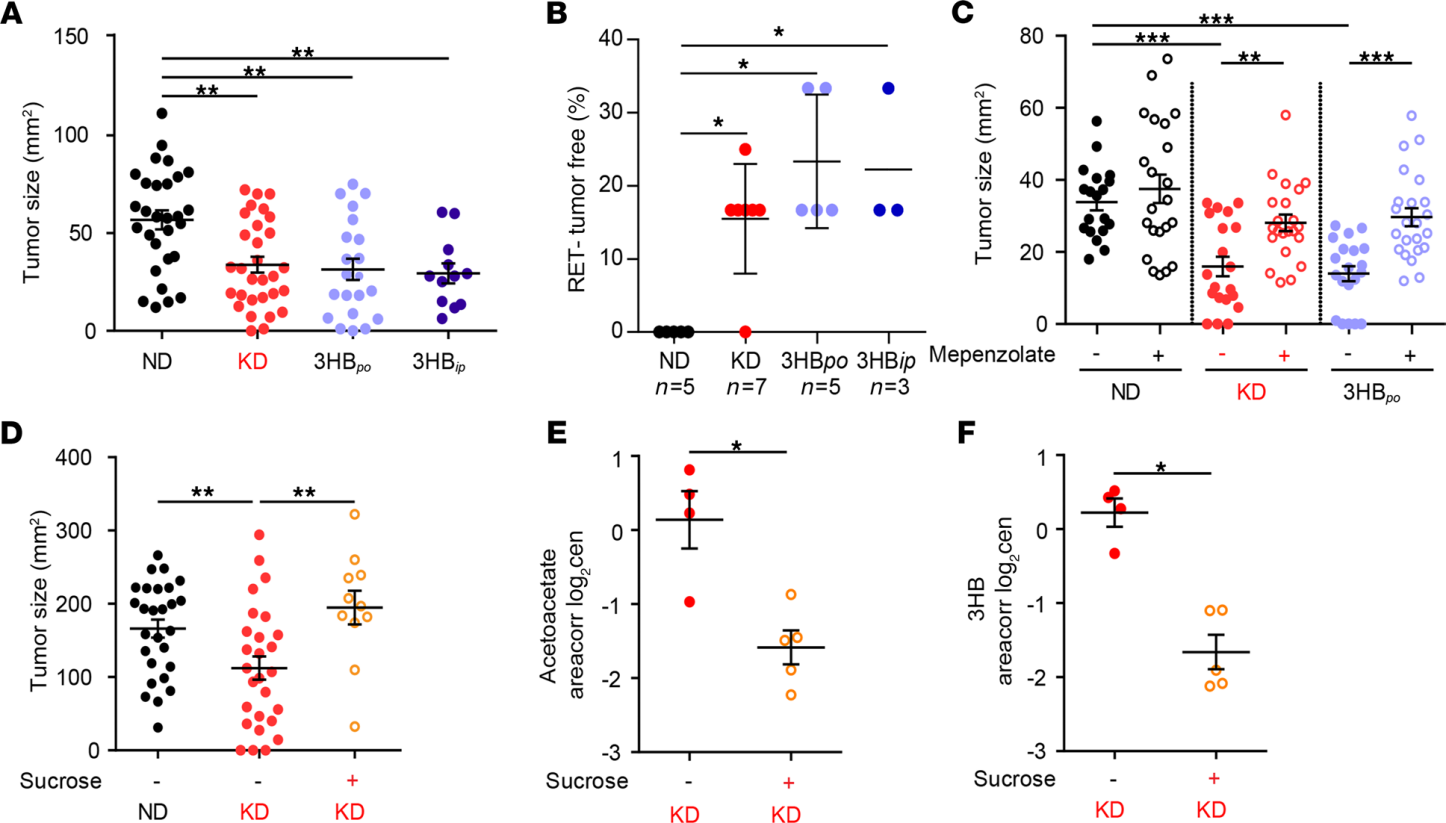
Ketone body 3-hydroxybutyrate (3HB) is necessary and sufficient to account for the anticancer effects of ketogenic diet. (**A** and **B**) Similar experimental setting as in Figure 1, A and B, comparing RET tumor size at day 12 of each nutritional intervention (normal diet [ND], ketogenic diet [KD], 3-hydroxybutyrate per os [3HB_po_] versus 3-hydroxybutyrate i.p. [3HB_ip_]) for all 12–15 mouse tumors from 3 independent experiments (**A**) and the complete regression rates for all the experiments performed (*n*, number of independent experiments encompassing 5–6 mice/group, SEM of percentage of tumor free mice across 3–7 experiments) (**B**). (**C**) Pharmacological inhibition of GPR109A (versus PBS as control) using i.p. daily administration of mepenzolate bromide (C21H26BrNO3). Tumor sizes at day 12 are depicted for each group. Data from 2–3 experiments involving 5–6 mice/group are depicted (*n* = 20 for ND, KD, and 3HB_po_ without mepenzolate and *n* = 24 for ND, KD, 3HB_po_ + mepenzolate. (**D**–**F**) Effects of sucrose supplementation on the antitumor (**D**) and metabolic effects (**E** and **F**) mediated by KD. Id. as in Figure 1, A and B, showing RET tumor sizes at day 12 after various dietary interventions (**D**) and the 2 ketone body (KB) plasma metabolites as in Figure 2, D and E (**E** and **F**). Results from 1 representative experiment out of 2 are depicted (*n* = 30 for group without sucrose and *n* = 12 with sucrose groups). Global comparison using Kruskall-Wallis test, with a post hoc multiple comparison using Dunn’s test (**A**–**D**); Student’s *t* test (**E** and **F**) (*P < 0.05, **P < 0.01, ***P < 0.001).

Importantly, the plasma levels of 3HB, but not those of acetoacetate, negatively correlated with the size of RET melanomas (Figure 2F). Driven by this correlation, we attempted to establish a cause-and-effect relationship between systemic KB and KD-mediated anticancer effects. For this, we replaced KD by administration of 3HB per os (3HB_po_). While an established protocol (4.2% 3HB drinking water) (36) caused a modest increase in plasma 3HB (0.5 and 1 mM), a higher dose (12.6% 3HB) allowed to achieve KD-related serum levels ≥3 mM (Supplemental Figure 2). Of note, in healthy individuals, blood concentrations of total ketones are generally less than 0.5 mmol/L (equivalent to 52.05 mg/L of 3HB), whereas after 6–7 days, fasting blood ketone concentrations can be 5–7 mmol/L (equivalent to 520.5–728.7 mg/L of 3HB), which is called starvation ketosis (37). Blood ketone concentrations in diabetic patients with ketoacidosis may exceed 25 mmol/L (38, 39). At this dose of 12.6%, 3HB_po_ retarded RET melanoma growth as much as KD retarded this growth, both in terms of kinetics and complete responses (Figure 3, A and B). Similarly, when 3HB was injected systemically (i.p.) to obtain KD-like pharmacokinetics, it mimicked the antitumor effects of KD (Figure 3, A and B, and Supplemental Figure 2). Of note, blockade of the 3HB receptor, GTP coupled receptor 109A (GPR109A) by means of mepenzolate bromide (C_21_H_26_BrNO_3_) (40) abolished the control of tumor progression by 3HB_po_, as well as by KD (Figure 3C). Similarly, when KetoCal, the usual KD, was supplemented with sucrose (5% in the drinking water), the induction of KB (3HB and acetate) was prevented and the anticancer effect of KD was lost (Figure 3, D–F, and Supplemental Figure 3).

Altogether, we conclude that KD reduces the growth kinetics of aggressive orthotopic cancers as a stand-alone treatment modality, at least in part through the pharmacological activity of 3HB on its receptor GPR109A.

### Contribution of microbial shifts and T lymphocytes to KD-mediated tumor growth control.

**Figure 4:**
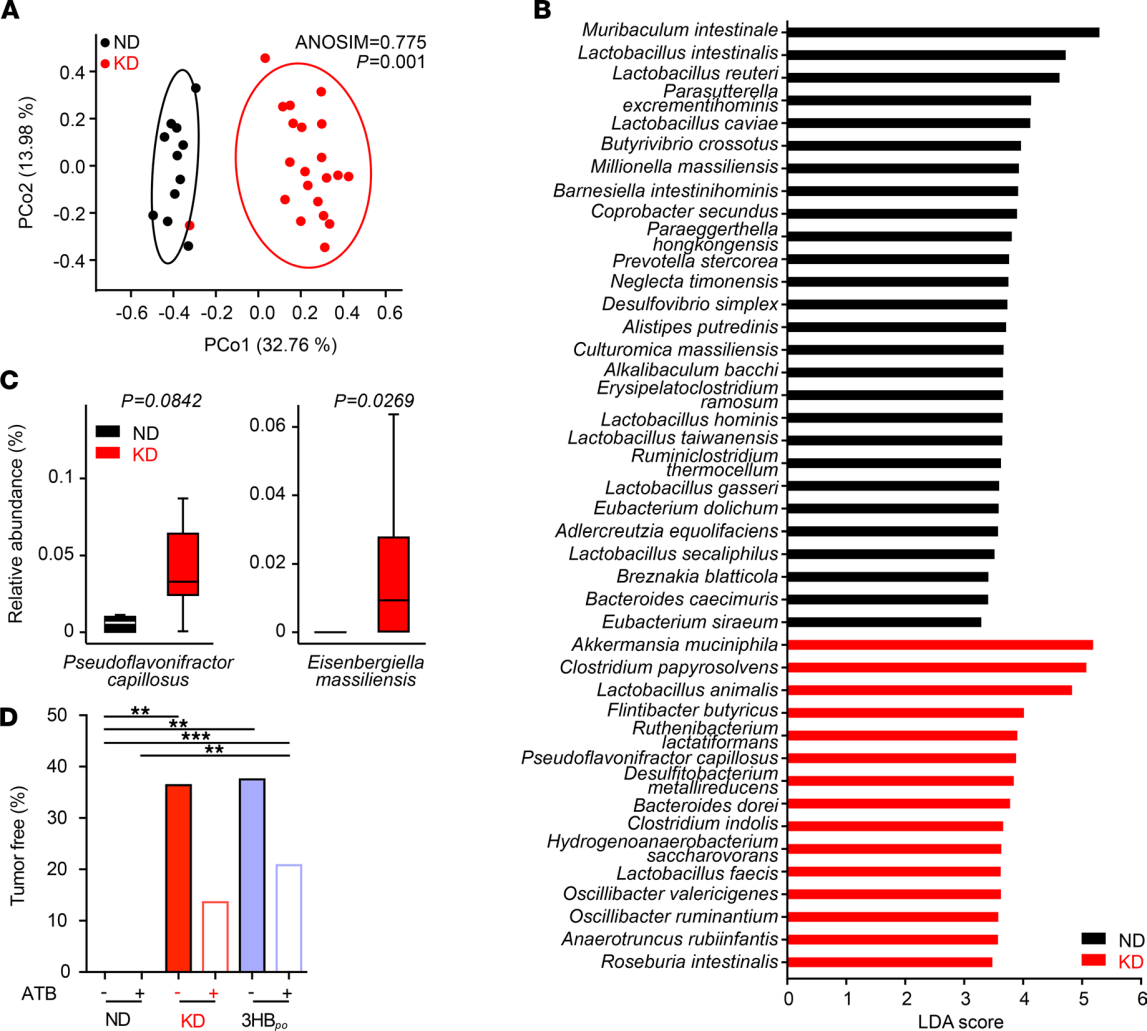
Ketogenic diet shifts the microbiota composition. (**A**) PCoA representing the differences in β diversity of fecal microbiota between dietary interventions normal diet (ND) versus Ketogenic diet (KD) at day 12 in RET–tumor bearing mice. ANOSIM and PERMANOVA defines the separation of the groups; the *P* value defines the significance of such separation after 999 permutations of the samples. (**B**) For each principal coordinate axis (PCo1 and PCo2), the collected variance, the Pearson *r* coefficient and the corresponding *P* value are shown. Partial Least Squares Discriminant Analysis (PLS-DA) plot of the variance between KD- and ND-fed tumor-bearing mice. LEfSe plot of species at day 12 discriminating ND- from KD-fed tumor-bearing mice, ordered by their LDA score. (**C**) Detailed relative abundance of distinct species. Refer to Supplemental Figure 4 for additional taxa. (**D**) Effects of broad-spectrum antibiotics on the percentages of tumor-free mice after ND or KD or 3-hydroxybutyrate per os (3HB_po_) after RET inoculation. Data from 2–3 experiments involving 5–6 mice/group are depicted (*n* = 12 for ND group and *n* = 21 for KD group). Student *t* test or ANOVA. **P < 0.01.***P < 0.001.

Oral intake of food or supplements can be expected to influence the intestinal microbiota. Indeed, the 16S rRNA sequencing of stools harvested at day 10 of KD in RET tumor bearers revealed significant deviations in the β diversity of operational taxonomic units (OTUs). Multidimensional principal coordinates analysis (PCoA) based on the Bray-Curtis Dissimilarity Index unraveled significant (*P* = 0.001, PERMANOVA test) compositional differences in ND versus KD samples (Figure 4A). Bacterial taxa with differential abundance between ND and KD groups were used as input for the linear discriminant analysis (LDA) to calculate an effect size (LEfSe analysis). KD induced an overrepresentation of *Akkermansia muciniphila*, *Ruthenibacterium lactatiformans*, and *Pseudoflavonifractor capillosus*, but a relative loss of more than 10 species belonging to the Lactobacillaceae family (Figure 4, B and C, and Supplemental Figure 4, A and B). When analyzing taxa of low prevalence that were not considered in the LEfSe analysis, we found that the relative abundance of *Eisenbergiella massiliensis* (Figure 4C, right panel) and *T. sanguinis* (Supplemental Figure 4B) were increased and decreased, respectively, in the stools of KD-fed mice, compared with ND.

**Figure 5:**
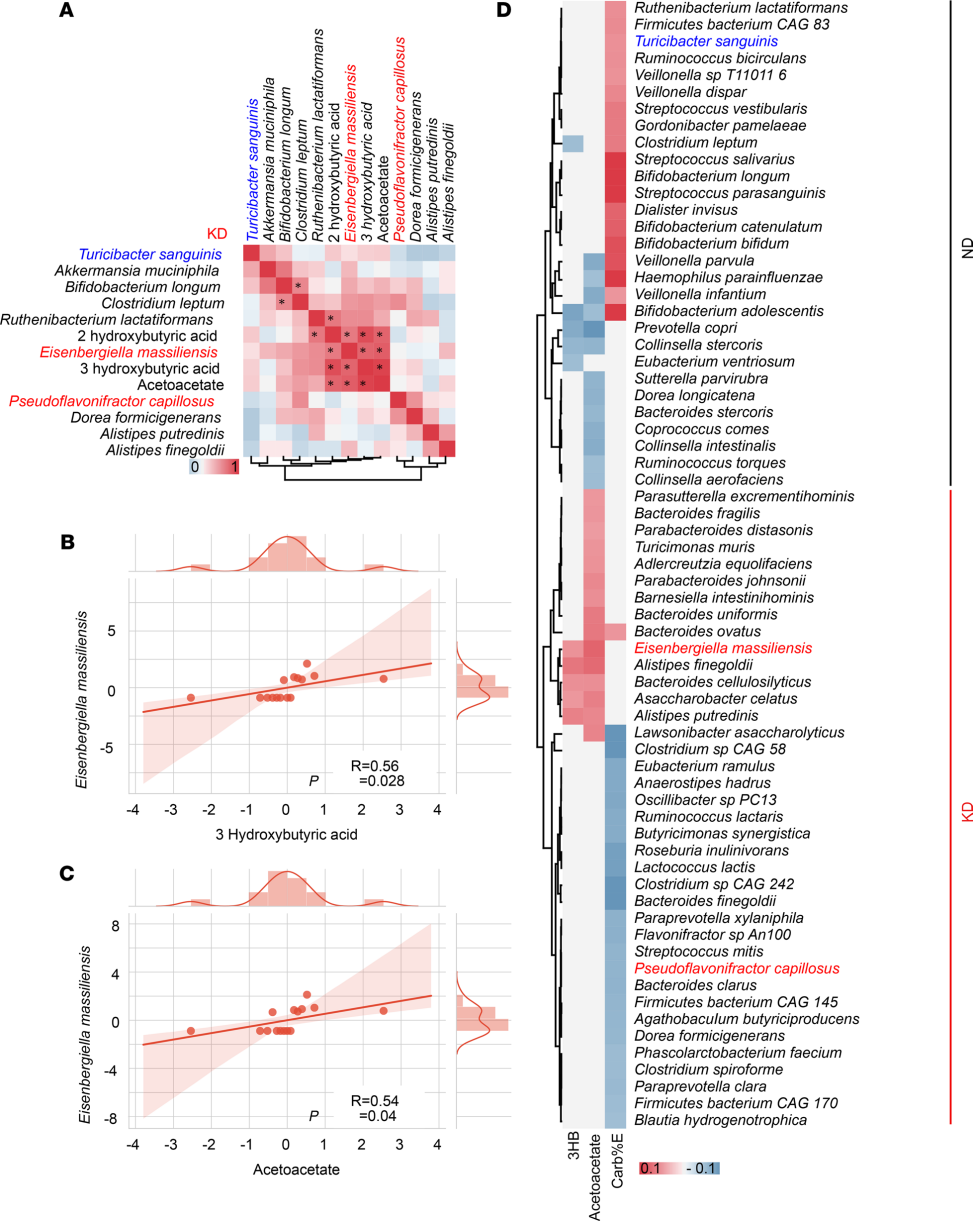
Correlations between microbial species and ketone bodies in mice and humans. (**A**–**C**) Mouse data. Alignment of the taxa relevant in our mouse preclinical studies and the plasma ketone bodies (KB) and Spearman correlations between 2 parameters. The significant ones are indicated with an asterisk (*P* < 0.05), and their raw data are presented in **B** and **C** (each dot represents 1 mouse). (**D**) Human data. Heatmap showing a correlation matrix between estimates of carbohydrate intake from food questionnaire (FQ) and KB monitoring in plasma in > 1000 individuals from the PREDICT-1 study. The taxa highly significant in our mouse models are highlighted. Pearson correlation was used.

We next assessed the effects of broad-spectrum antibiotics (ATB) on the anticancer activity of KD and 3HB. ATB tended — albeit not significantly — to reduce the protective effect of both nutritional interventions against RET melanoma outgrowth (Figure 4D). In a Spearman correlation matrix integrating all bacterial taxa and KB, we found that *Eisenbergiella massiliensis* was at the center of the KB-related metacluster (Figure 5A). Indeed, its relative abundance in feces correlated with the plasma concentrations of the 2 KB (acetoacetate and 3HB), only in KD-treated mice and not in ND controls (Figure 5, A–C, and Supplemental Figure 4C).

To analyze the clinical relevance of these findings, we turned to an epidemiological study linking food ingredients, systemic inflammatory markers and gut microbiota— the Personalized Responses to Dietary Composition Trial (PREDICT-1) — that enrolled 1000 participants (mono and dizygotic healthy twins from the Twins UK cohort, and nontwin healthy individuals). This trial consisted of collecting food questionnaires, plasma metabolomics, and shotgun metagenomics data (41). We analyzed correlations between plasma KB concentrations, carbohydrate consumption (using an estimate of carbohydrates normalized to energetic contribution; carb%E), and the intestinal OTUs identified in the murine KD fingerprint. Again*, E*. *massiliensis* positively correlated with 3HB and acetoacetate (*P* < 0.05) in humans, while *Bifidobacterium adolescentis* and *Prevotella copri* anticorrelated with plasma KB levels (Figure 5D). *Turicibacter sanguinis* and *P. capillosus* positively and negatively correlated with carb%E, respectively (Figure 5D).

**Figure 6:**
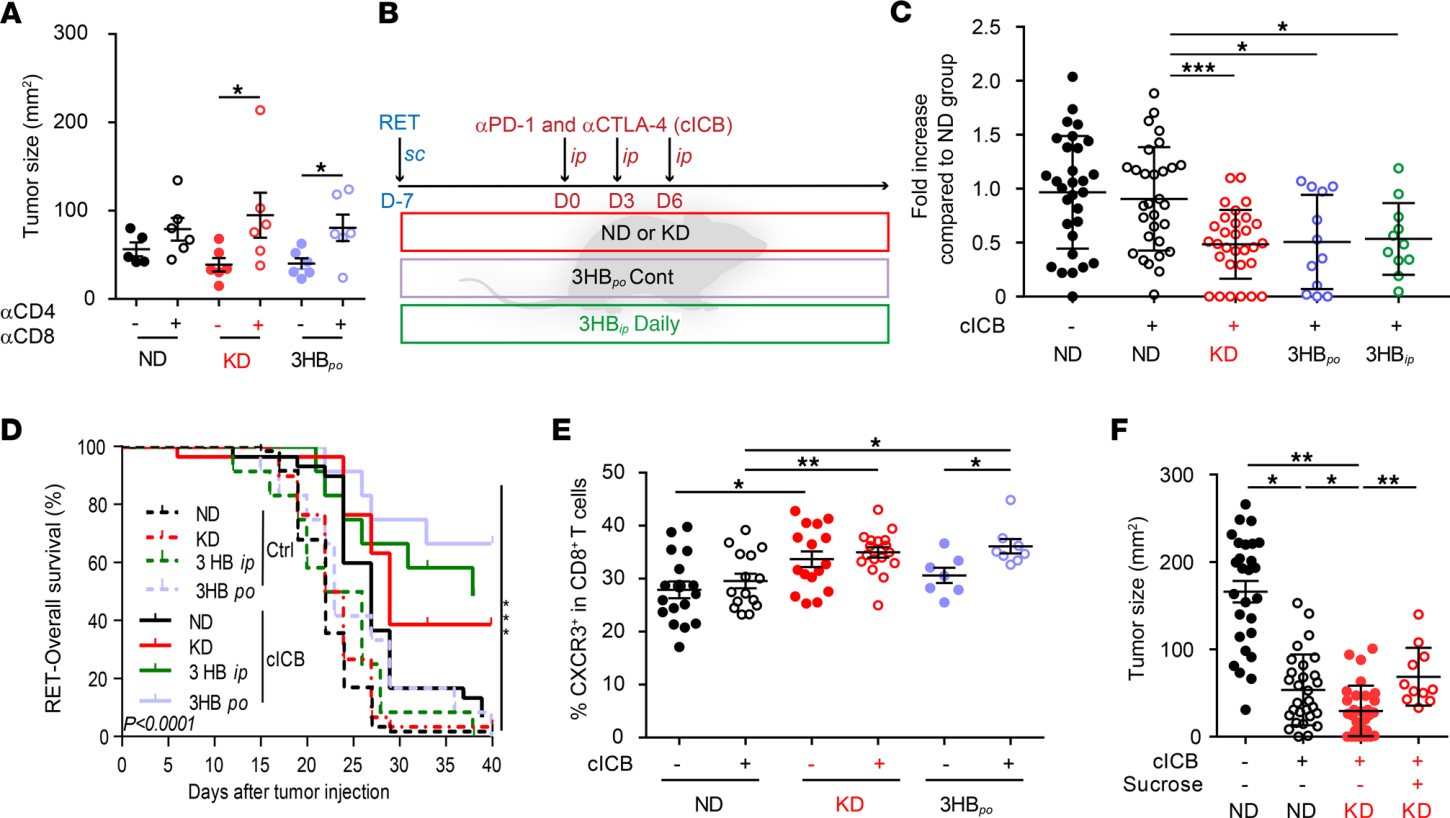
T cell–dependent effects of ketogenic diet and synergy with immune checkpoint blockade. (**A**) Effects of T cell depletion on tumor sizes at day 12 of diet interventions. Tumor sizes in all diet-fed (normal diet [ND], ketogenic diet [KD], or 3-hydroxybutyrate per os [3HB_po_]) groups in the presence of i.p. injections of anti-CD4 and anti-CD8 depleting Abs prior to tumor inoculation. A representative experiment out of 2 is depicted, both yielding similar conclusions; each dot represents 1 mouse, and each group contained 5–6 mice. Mann Whitney *U* statistical analyses was used. *P < 0.05. (**B**) Experimental setting for combinatorial regimen. (**C** and **D**) Effects of diet interventions on tumor size (**C**) at day 9 after RET inoculation and diet intervention, after only 1 i.p. administration of cICB (anti–CTLA-4 plus anti–PD-1 mAbs) and overall survival (**D**). Results of 4 concatenated experiments are depicted; each dot represents 1 mouse (*n* = 30 for ND and KD groups; *n* = 12 for 3HB_po_ and 3HB_ip_). (**C**) Fold increase of tumor sizes in cICB groups fed with ND, KD, or 3HB_po_ or 3-hydroxybutyrate i.p. (3HB_ip_) (normalized to ND groups). (**D**) Overall survival appreciated with Kaplan Meier curves for 12 animals/group after a complete treatment combining continuous diet plus cICB, according to **B**. (**E**) Flow cytometry analyses of Tc1 (defined as CXCR3^+^CD8^+^ T splenocytes) at day 9 of the combinatorial regimen; each dot represents 1 spleen. Results from 2 pooled experiments are shown. (**F**) Effects of sucrose supplementation on the synergy between diets and cICB against RET tumor progression. Individual tumor sizes at day 19 of 3 concatenated experiments involving 6 mice/group are shown for each diet intervention; each dot represents 1 tumor/mouse (*n* = 27 for groups without sucrose, *n* = 28 for groups on sucrose). Student’s *t* test (**A**); global comparison using Kruskall-Wallis test and post-hoc multiple comparisons using Dunn’s test (**C** and **E**–**F**). *P < 0.05, **P < 0.01, ***P < 0.001.

Given the reported immunostimulatory capacity of distinct OTUs selected from the KD (such as *A*. *muciniphila*) (42), we next analyzed the effects of T cell depletion on the tumor growth–reducing effect of KD and 3HB_po_. Both nutritional interventions lost their anticancer effects in mice injected with antibodies against CD4 and CD8 (Figure 6A).

In conclusion, KD and 3HB mediate their antitumor activity not through direct, cancer cell–autonomous mechanisms, but rather through effects on the host immune system.

### Synergistic antitumor effects of ketogenic interventions with ICB.

Driven by the consideration that KD or 3HB induce T cell–dependent anticancer effects, we combined these nutritional interventions with the most powerful combination of immunostimulatory mAbs in the clinics, thus targeting both PD-1 and CTLA-4. In the RET melanoma model, diet-based host conditioning increased the efficacy of combination ICB (cICB; anti–PD-1 plus anti–CTLA-4), with significant differences observed as early as after the first systemic injection of cICB (Figure 6, B and C). In the context of complete cICB regimen (3 i.p. injections over a 6-day period), 3HB_po_ boosted the tumor growth–inhibitory activity of cICB, prolonging overall survival of almost 60% of RET tumor bearers (Figure 6D). 3HB_po_ was superior to KD, although both nutritional interventions led to an increase in the cICB-induced pool of type 1 CD8^+^ T cells (Tc1) (CXCR3^+^CD8^+^) splenocytes (Figure 6E). Of note, both diet interventions tended to mobilize the recirculation of patrolling activated monocytes by day 5 (Supplemental Figure 5, C–E). The improvement of cICB-induced tumor growth reduction conferred by KD was lost upon supplementation with sucrose, which breaks ketogenesis (Figure 6F).

**Figure 7:**
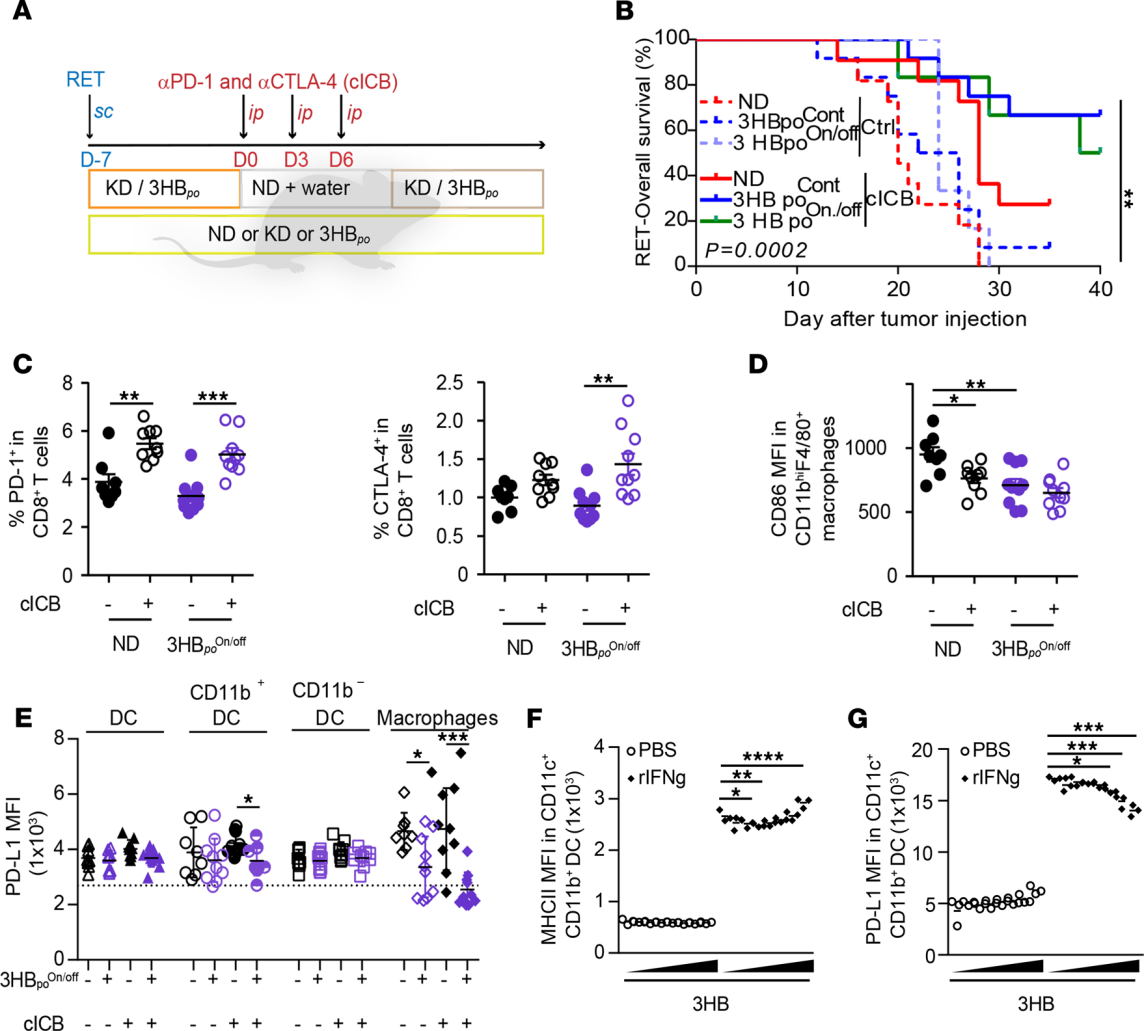
Intermittent 3HB scheduling affects systemic expression of T cell inhibitory receptor and their ligands. (**A** and **B**) Experimental setting for intemittent diet and combination immune checkpoint blockers (cICB) in the RET model and Kaplan Meier curves of overall survival for 12 animals/group, according to therapeutic scheme comparing continuous (Cont) versus intermittent (On/off) diet interventions. (**C**) Flow cytometric analyses of PD-1 and CTLA-4 expression in splenic CD8^+^ T lymphocytes at day 17, after diet (ketogenic diet [KD] or 3-hydroxybutyrate [3HB] Cont or On/off) and systemic cICB combinatorial regimen in RENCA bearing BALB/c mice. Refer to Supplemental Figure 5, A and B, for Tim-3, Lag-3, and 4-1BB in all splenic T cells. (**D** and **E**) Flow cytometry analysis of MFI for the membrane expression of costimulatory (CD86 in **D**) or inhibitory (PD-L1 in **E**) on macrophages (**D**) and other myeloid subsets from the spleens at day 17, after complete diet (3HB On/off) and systemic cICB combinatorial regimen in RENCA-bearing BALB/c mice. The results from 2–3 experiments involving 6 mice/group are depicted; each dot represents 1 spleen. (**F** and **G**) In vitro effects of increasing dosing of 3HB onto a DC cell line stimulated or not with rIFN-γ. Flow cytometry determination of MHC II/I-Ab (**F**) and PD-L1 expression (**G**) as mean fluorescence intensity (MFI) on the surface expression at 48 hours of stimulation. One representative experiment out of 4 is depicted, yielding similar conclusions (*n* = 12 or each groups). Student’s *t* test (**C**), global comparison using Kruskall-Wallis test with post hoc multiple comparisons using Dunn’s test (*P < 0.05) (**D** and **E**). Global comparison using ANOVA with post hoc multiple comparisons using Dunnett’s test (*P < 0.05) (**F** and **G**). *P < 0.05, **P < 0.01, ***P < 0.001.

Given that most nutritional interventions are prescribed in intermittent courses to limit the constraints on the users, we compared continuous versus discontinuous (1 week on, 1 week off) regimens of the KD or 3HB_po_ (Figure 7, A and B) in the RET tumor model. Surprisingly, the combined effect of 3HB_po_ and cICB tended to be favored by the intermittent schedule (Figure 7B). In this latter setting, flow cytometry analyses of splenocytes revealed that the On/off regimen of 3HB_po_ alone decreased the expression of activation/exhaustion markers (such as CTLA-4 and CD223, best known as Lag-3) on CD4^+^ T cells (Supplemental Figure 5A), and prevented the cICB-induced upregulation of Lag-3 and CD137 (best known as 4-1BB) on CD8^+^ T cells (Supplemental Figure 5B). In contrast, the combination of cICB and intermittent 3HB_po_ boosted the expression of CTLA-4 on CD8^+^ T cells but simultaneously reduced the expression of CD86, the CTLA-4 ligand, on splenic myeloid cells (Figure 7, C and D). Conversely, while PD-1 remained stable, the expression of its ligand PD-L1 was significantly decreased on splenic macrophages during the combinatorial therapeutic regimen (Figure 7E). To confirm that 3HB_po_ contributes to restrain PD-L1 expression despite the presence of a pool of Tc11 cells producing the PD-L1 promoting cytokine IFN-γ in vivo (43), we exposed BM-derived myeloid antigen–presenting cells to 3HB prior to exposure to recombinant IFN-γ (rIFN-γ) in vitro. Indeed, low (micromolar) concentrations of 3HB reduced the IFN-γ–induced upregulation of PD-L1 membrane expression but not that of major histocompatibility complex class II molecules (MHC II) (Figure 7, F and G). Of note, 3HB did not modulate IFN-γ–induced upregulation of PD-L1 membrane expression on tumor cells (Supplemental Figure 5C). In summary, when combined with ICB, ketogenic regimens (KD or 3HB) induced the splenic accumulation of CXCR3^+^ Tc1 and induced the upregulation of PD-1 and CTLA-4 on CD8^+^ T cells, but it prevented the expression of their ligands on splenic macrophages, setting the stage for prolonged systemic T cell activation.

**Figure 8:**
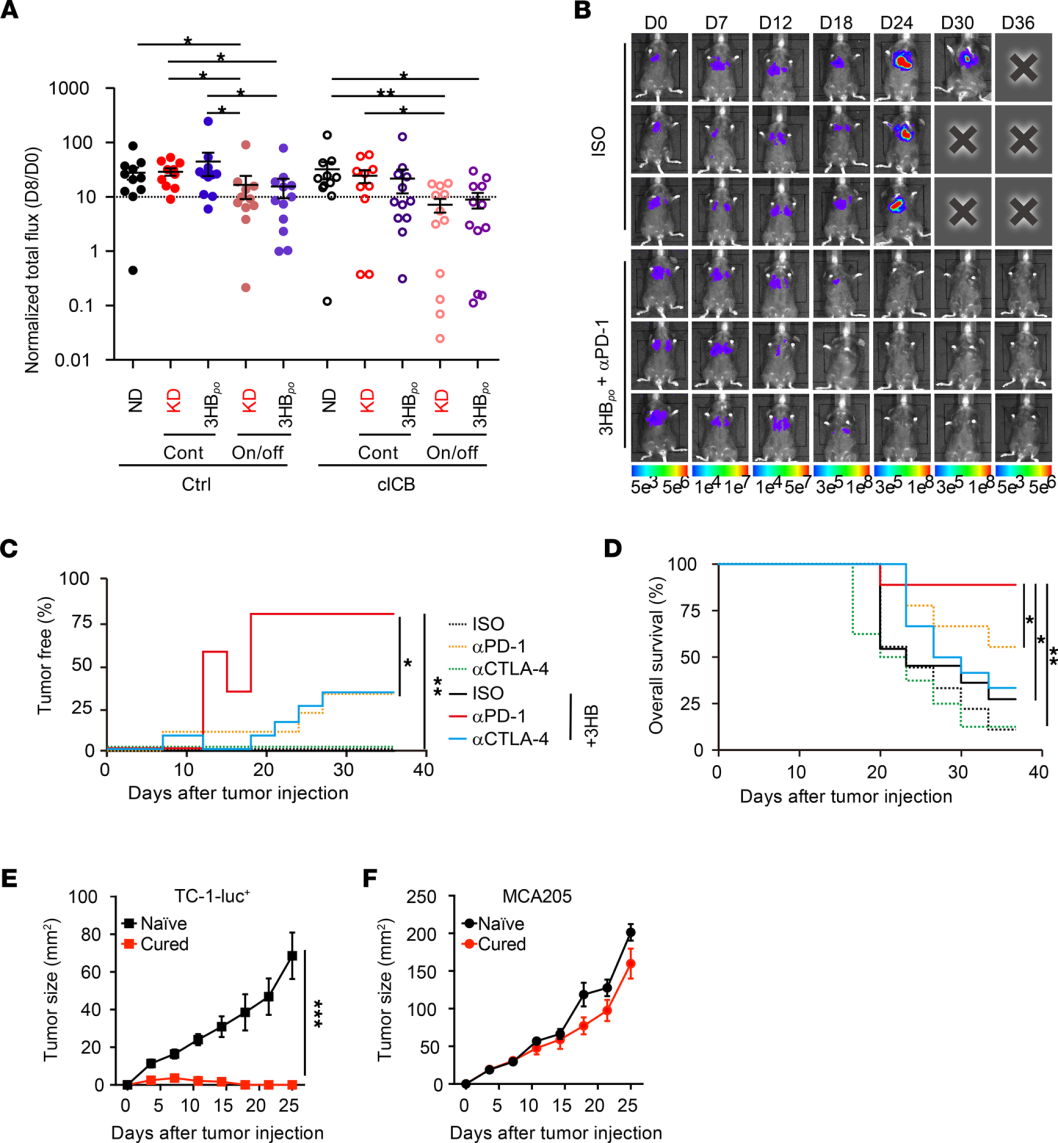
Efficacy of intermittent 3HB in circumventing primary resistance to PD-1 blockade in an orthotopic cancer model. (**A**–**F**) Extension of the comparison between continuous (Cont) versus intermittent (On/off) feeding with KD or 3HB_po_ in 2 tumor models (RENCA [**A**], TC-1 [**B**–**D**]) treated with various therapeutic mAbs (refer to Supplemental Figure 6, A and B, for experimental setting designs). Cross-sectional RENCA tumor burden monitored by bioluminescence imaging of luciferase activity (ratio of luminescence at D8 versus D0 [D8: day of randomization]) (**A**) are depicted, gathering 2 independent experiments containing 6 mice/group. All experiments were performed twice. Monitoring of TC-1 progression using bioluminescence imaging (**B**) and percentages of TC-1_luc tumor–free animals overtime monitored by bioluminescence imaging of luciferase activity (**C**) and overall survival (**D**) estimated by Kaplan Meier curves for 2 experiments of 6 mice/group pooled together. (**E** and **F**) Tumor growth curves represented as mean ± SEM of tumor sizes for each group after s.c. rechallenge of TC-1_luc tumor–free mice (from **B**–**D**) with the MTD of TC-1_luc (**E**) or irrelevant MCA205 (**F**) (refer to Supplemental Figure 6C for experimental setting designs). Experiments were performed twice (*n* = 12 per groups). Global comparison for the log_10_-normalized data using ANOVA with post hoc multiple comparisons using Sidak’s test (*P < 0.05) (**A**) or dedicated software (https://kroemerlab.shinyapps.io/TumGrowth/) (**C**–**F**). *P < 0.05, **P < 0.01, ***P < 0.001.

We next turned to orthotopic models of established carcinomas to evaluate the efficacy of KD in the context of ICB. Established RENCAs were best controlled when their BALB/c hosts were treated with cICB plus KD or 3HB, but only when the nutritional interventions were applied intermittently (Figure 8A and Supplemental Figure 6A). We next selected an aggressive orthotopic TC-1 lung cancer tumor model, which is primarily resistant to anti–PD-1 or anti–CTLA-4 Abs, as stand-alone therapies (Supplemental Figure 6B). Importantly, the only efficient regimen that led to the eradication of established tumors (in > 70% of the cases), allowing for long-term survival of most of the C57BL/6J hosts, was the combination of intermittent 3HB_po_ and anti–PD-1 mAbs (Figure 8, B–D). Mice that had been diagnosed with TC-1 lung cancers (by bioluminescence imaging) and then cured by the combination of 3HB_po_ and anti–PD-1 resisted s.c. rechallenge with TC-1 cancers, yet readily developed MCA205 fibrosarcomas, indicating that they developed a specific long-term protective immune response (Figure 8, E and F, and Supplemental Figure 6C).

Altogether, these results support the idea that ketogenic regimens can enhance the anticancer effects of ICB in multiple orthotopic tumor models, including melanoma, renal, and non–small cell lung cancer.

## Discussion

So far, the rationale to use KD for combating neoplasia was based on a cell-autonomous hypothesis. Here, we highlight a non–cell autonomous mechanism of action of KD that can be exploited to ameliorate anticancer therapies. KD — and, more specifically, 3HB — induce immunostimulatory effects in secondary lymphoid organs that are important for natural and therapy-induced immunosurveillance.

Oral administration of 3HB increased the expansion of CD8^+^ T cells elicited by ICB, while restraining the expression of activation/exhaustion markers such as CTLA-4, Lag-3, and 4-1BB/CD137 in the spleen. Concomitantly, 3HB limited the IFN-γ–induced PD-L1 and CD86 expression on splenic macrophages in vivo (for both) and in vitro (for PD-L1), reducing a negative feedback signal operating after T cell receptor (TCR) engagement. The poor expression of PD-1 and CTLA-4 receptor ligands may maintain T cell activation and fitness in secondary lymphoid organs. Of note, pioneering work highlighted the pivotal role of PD-L1 expression by antigen presenting cells in the efficacy of PD-1 blockade (44–46), suggesting that KD may be pivotal to prime the host for a full-blown immune response to immune checkpoint inhibition. One single report (47) indicated the capacity of KD to elicit systemic innate immune responses through IL-17–producing γδ T cells during virus infection. However, in this report (47), KB — including 3HB — could not substitute for KD to stimulate antiviral immunity. This is different from our observation that KD and 3HB have similar immune-dependent antitumor effects. Moreover, in tumor-bearing mice, we failed to observe any effects of KD on γδ T and NK innate effectors (data not shown).

Importantly, KD and 3HB slowed natural tumor progression in the absence of additional therapeutic intervention, but they also accelerated and improved the efficacy of cICB against established and aggressive orthotopic melanoma, lung, and renal cell cancers. This anticancer effect was blunted by sucrose supplementation (in the case of KD) or by blocking the 3HB receptor (for both KD and 3HB). Indeed, T cells and GPR109A engagement were mandatory for the full-blown anticancer properties of 3HB, paving the way to the accelerated efficacy of ICB.

As expected, KD markedly affected the composition of the gut microbiota, shifting the balance from tolerogenic (Lactobacilli spp., *C*. *asparagiforme*) toward immunogenic bacteria (such as *A*. *muciniphila*) (48). Of note, immunogenic bacteria can share antigens with oncogenic drivers and boost the cognate T cell arm of immune responses (49). However, several arguments indicate that the modulation of the microbiota does not play a central role in the anticancer effects of KD. First, antibiotics did not significantly compromise the anticancer efficacy of the KD or 3HB. Second, the i.p. administration that mimics parenteral administration in mice of 3HB recapitulated the findings obtained with KD. Third, we failed to observe additive effects of prebiotics and probiotics in the same cancer models (not shown). All these lines of evidence argue in favor of a direct (microbiota-independent) immunostimulatory action of KD.

Interestingly, our data appear to indicate that intermittent administration of 3HB might be more beneficial than the continuous regimen, alone or in combination with cICB. A large body of literature reports that intermittent fasting, which leads to discontinuous ketogenesis, is associated with improved health (50–52). Moreover, intermittent KD can improve the health span of mice more efficiently than continuous KD (53). Indeed, cyclic KD may prevent high-fat diet–associated obesity, resulting in long-term maintenance of normal weight, and cyclic KD resulted in higher plasma levels of β-hydroxybutyrate than continuous KD, particularly during daytime (53). More work will be needed to delineate the immunological and/or metabolic mechanisms underpinning these effects in cancer patients.

Regardless of these limitations, the present work has important implications, as previously discussed, not only for obese individuals and athletes, but also in brain and endometrial cancer patients (54–56). KD might be implemented prior, or concomitantly, to anticancer therapies (including immunogenic chemotherapy and immunotherapy) with the scope of potentiating their immunostimulatory effects, reducing the number of therapeutic cycles, and achieving a higher rate of complete cures. Given the poor palatability of KD and the consequent lack of compliance, as well as the potential toxicity of KD on the cardiovascular system (57), substitution of KD by its main metabolite 3HB represents an attractive option. Numerous clinical trials are underway to evaluate KD either as a stand-alone cancer treatment, for instance in children with brain tumors (NCT03328858, NCT03955068) or adults with advanced cancers (NCT01716468), or in association with other treatments such as chemotherapy (NCT03535701) and hormonotherapy (NCT03962647) in breast cancers, as well as chemoradiation in lung cancers (NCT01419587). Only 1 trial is investigating the effect of a ketogenic drink on perioperative and oncologic outcomes in cancer patients (NCT03510429; https://clinicaltrials.gov/).

Prospective randomization and appropriate pharmacodynamics markers will be necessary to adjust dosing and scheduling, as well as the clinical endpoints of these studies. Our findings emphasize that not only metabolic and inflammation-related parameters, but also immunological parameters, must be evaluated to appreciate the impact of nutritional interventions on cancer.

## Methods

### Mice.

Female C57BL/6JOlaHsd and BALB/cJRj were purchased from Harlan and Janvier, respectively. Mice were used between 8 and 16 weeks of age. All mice experiments were performed at Gustave Roussy Cancer Campus, and mice were housed in SPF conditions or maintained in isolators.

### Cell lines.

RET melanoma (transgenic expression of the Ret protooncogene) under the control of the metallothionein-1 promoter driving spontaneous melanogenesis was provided by Viktor Umansky (DKFZ — German Cancer Research Center; syngeneic from C57BL/6J mice). The luciferase-expressing TC-1 cell line (TC-1_luc) was shared by Tzyy-Choou (The Johns Hopkins Hospital, Baltimore, Maryland, USA) (58); luciferase-transfected RENCA cell lines (syngeneic for BALB/c mice, provided by Transgene) were cultured at 37°C under 5% CO_2_ in RPMI-1640 medium supplemented with 10% heat-inactivated FBS, 1% penicillin/streptomycin, 2 mM L-glutamine, 1% of sodium pyruvate, and nonessential amino acids (all from Invitrogen), hereafter referred to as complete RPMI medium. Cell lines were regularly tested for mycoplasma contamination, and cells were not used for more than 10 passages.

### Diet.

Mice were randomized according to their weight before the beginning of the diet and assigned to control ND, control KD, 3HB per os (3HB_po_), or i.p. 3HB (3HB_ip_) groups.C57BL/6J mice started their diet the very day of s.c. inoculation with RET. BALB/c mice were fed different diets — ND, KD, or 3HB_po_ — 10 days before the RENCA inoculation into the sucapsular area of the kidney. The KD, KetoCal, was purchased from Nutricia. KetoCal is a nutritionally complete ketogenic formula with a ketogenic ratio (fats/proteins plus carbohydrates) of 4:1. The fat was derived from soybean- oil. The KetoCal diet was fed to mice in a paste form (water/KetoCal; 1:2) within the cage (59). We freshly prepared the mixture corresponded to 10 g of KD per day. The diets were changed 3 times a week. Mice in the 3HB_po_ groups received ND in food, but drinking water was replaced by ad libitum Ketoforce, purchased from Ergomax (59). We freshly prepared the mixture at 10 g of KD per day. The diets were changed 3 times a week. Mice in the 3HB_po_ groups received ND in food, but drinking water was replaced by ad libitum Ketoforce (purchased from Ergomax) (36), which was mineral salts 3HB solution at a final concentration of 4.2 mL in 100 mL of water. The concentration corresponds to 16.38 g of 3HB in 100 mL of water. 3HB_po_ solution was changed 3 times a week to maintain the stability of the product during the entire experiment. Mice in 3HB_ip_ groups received ND in food and regular drinking water but were injected i.p. with β-hydroxybutyric acid sodium salt purchased from MilliporeSigma. We prepared extemporaneously the 3HB_ip_ at 178 mg/mL in PBS 1×. The injections were performed daily during 8 days every other week (the On/off protocol).

For animals inoculated with TC-1_luc cells, treatment of 3HB (3× = 12% KetoForce in drinking water) was initiated 9 days before tumor inoculation, in an On/off dotted mode (3-day 3HB solution/3-day water). After TC-1 injection, the treatment of 3HB was maintained in the On-off system during the whole experiment. For the experiments aimed at interrupting ketosis, sucrose (C12H22O11, MilliporeSigma) was added in the water at a final concentration of 5% in drinking water. The bottle was changed 3 times a week to avoid contamination.

### s.c. RET melanoma model.

C57BL/6J mice were s.c. injected in their flank with 0.5 × 10^6^ RET cells. Tumor size was routinely monitored every 3 days using a caliper. When tumors reached 28–30 mm² in size, tumor-bearing mice were randomized to receive a cICB — i.e., neutralizing mAbs anti–PD-1 (clone RMP1-14, 250 μg/mouse) and anti–CTLA-4 (9D9, 100 μg/mouse; Bio X Cell). Mice were injected i.p. with cICBs or combined isotype control antibodies (clone 2A3 and MPC11) 3 times at 3-day intervals. All mAbs and the recommended isotype control mAbs for in vivo use were obtained from BioXcell.

### RENCA-luc orthotopic kidney cancer model.

The day of tumor challenge, mice were anesthetized with isofluorane; then, 1 × 10^4^ RENCA tumor cells resuspended into 30 μL of PBS were injected into the subcapsular space of the right kidney. The skin incision was then closed with surgical clips. Tumor incidence (at day 7) and development were monitored by in vivo photonic imaging of tumor cell luciferase activity. Briefly, mice received an i.p. injection of the substrate of luciferase (E1605, Promega) at a dose of 3 mg/kg, and in vivo photonic imaging was acquired with an IVIS Spectrum Imaging Series (PerkinElmer). Tumor-bearing mice were randomized to designed groups for treatments with PD-1 or CTLA-4 blocking antibodies, or equivalent isotype control antibodies, at day 7 after tumor challenge (as mentioned previously). Tumor growth was monitored once weekly.

### TC-1_luc orthotopic lung cancer model.

To establish the orthotopic lung cancer model at day 0, TC-1_luc cells (5 × 10^5^/mouse, in 100 μL PBS) were i.v. injected to WT C57BL/6J mice (8-week-old female). Tumor incidence was monitoring at day 10, and mice were randomized between ICB- or cICB-treated groups or isotype control Ab groups. Photonic imaging was acquired on a Xenogen IVIS 50 bioluminescence imaging system for TC1 model. Mice were injected i.p. with anti–PD-1 (29F.1A12, 200 μg/mouse), anti–CTLA-4 (9D9, 100 μg/mouse), cICB or isotypes controls The ICB treatment was administrated 4 times with 4-day intervals. In vivo imaging was acquired every 3–5 days, with the exposure time of 4 minutes, with the appropriate exposure time if saturation occurred. 3HB dosing was 3× the dosing utilized for RET, given the pharmacokinetics depicted in Supplemental Figure 2C.

### T cell depletion.

T cell depletion was performed by i.p. treatment with depleting anti-CD4 and anti-CD8 mAbs (GK1.5 and 53–6.72; 200μg/mouse) or respective isotype controls (LTF-2 and 2A3). Depletion treatment started 4 days before RET challenge and repeated at the same dose every 7 days. In each group, tumor growth was monitored 3 times per week.

### Antibiotic treatments.

Mice were treated with an antibiotic solution (ATB) containing ampicillin (1 mg/ml), streptomycin (5 mg/ml), and colistin (1 mg/ml), with or without the addition of vancomycin (0.25 mg/ml) via the drinking water (all purchased from MilliporeSigma). Solutions and bottles were replaced 3 times and once weekly, respectively. In brief, mice were treated with ATB throughout the experiment, with the addition of vancomycin for the first 4 days with ATB and without the addition of vancomycin continuously throughout the experiment from then on. Antibiotic activity was confirmed by cultivating fecal pellets resuspended in PBS 1× at 0.1 g/mL on COS plates (5% Sheep Blood Columbia Agar; bioMérieux) plates for 48 hours at 37°C in aerobic and anaerobic conditions.

### Feces and plasma collection.

Plasma and feces were harvested in each mouse and group for metagenomics and metabolomics, respectively, at day 10, at the day of RET inoculation, or after completion of immunotherapy. Blood was drawn in EDTA tubes after mouse anesthesia in the submandibular area; it was then centrifuged 10 minutes at 5000*g* to allow plasma harvesting at room temperature. Samples were stored at –80°C until processing.

### Metabolomics sample preparation.

About 30 mg of tissue for each condition was first weighted and solubilized into 1.5 mL microtubes with ceramic beads, with 1 mL of cold lysate buffer with ISTD (MeOH/Water/Chloroform, 9/1/1, -20°C); it was then homogenized and centrifuged (–8°C, 10 minutes, 5000*g*). Then, the upper phase of the supernatant was split in 3 parts: gas chromatography–mass spectrometry (GC-MS), short-chain fatty acids (SCFA) and KB monitoring, and reversed phase UHPLC-MS. The rest of the supernatant was spiked with methanol (2% of sulfosalicylic acid), centrifuged (–8°C, 10 minutes, 5000*g*), evaporated, and recovered with MilliQ water before injection in UHPLC/MS of the polyamines method. The GC-MS aliquot was evaporated, and metabolites were derivatized with methoxyamine in pyridine and N-Methyl-N-(trimethylsilyl) trifluoroacetamide (MSTFA). SCFA and KB were derivatized with 3 nitrophenylhydrazine (3-NPH) and N-(3-Dimethylaminopropyl)-N′-ethylcarbodiimide hydrochloride (EDC). Reverse-phase LC-MS aliquots were evaporated and solubilized with MilliQ water. Regarding plasma preparation, a volume of 25 μL was mixed with 250 μL of same cold solvent mixture as above into a 1.5 mL microtube and prepared in a similar way as tissues. The GC-MS/MS method was performed on a 7890B gas chromatography (Agilent Technologies) coupled to a 7000C Triple Quadrupole (Agilent Technologies) equipped with a High sensitivity electronic impact source (EI) operating in positive mode. Helium gas flowed through column (J&W Scientific, HP-5MS, 30 m × 0.25 mm, internal diameter [i.d.] 0.25 mm, Agilent Technologies Inc.) at 1 mL/min. Targeted analyses of bile acids and polyamines were performed separately on a RRLC 1260 system (Agilent Technologies) coupled to a 6410 Triple Quadrupole (Agilent Technologies) equipped with an electrospray source. The collision gas was nitrogen. The scan mode used was the multiple reaction monitoring (MRM). Peak detection and integration of the analytes were performed using the Agilent Mass Hunter quantitative software (B.07.01). Targeted analysis of SCFA and KB were performed on a RRLC 1260 system (Agilent Technologies) coupled to a 6500+ QTRAP (Sciex) equipped with an electrospray ion source. The instrument was operated using MRM. The software used to operate the mass spectrometer was Analyst (Version 1.7). Peak detection, integration, and quantification of the analytes were performed using MultiQuant quantitative software (Version 3.0.3). The profiling experiment was performed with a Dionex Ultimate 3000 UHPLC system (Thermo Fisher Scientific) coupled to a Q-Exactive (Thermo Fisher Scientific) equipped with an electrospray source operating in both positive and negative mode and full scan mode from 100 to 1200 *m/z*. The mass spectrometer was calibrated with sodium acetate solution dedicated to low mass calibration. Peak detection and integration were performed using the Thermo Xcalibur quantitative software (Version 2.2).

### Calibration curve preparation of 3HB acid in plasma (absolute quantification).

Since basal concentrations of 3HB can be monitored in the plasma of animals prior to any dietary intervention, the metered addition method was used for absolute quantification analysis. To prepare calibration samples, stock solution of 3HB was diluted in 25 μL of mice plasma (lithium heparin) to obtain 6 calibration points with concentrations of 46.49, 93.36, 237.06, 464.92, 933.57, and 9335.7μM. The calibration curve was prepared the same day and treated the same way as the SFCA and KB method sample aliquots: after protein precipitation, 40 μL of the supernatant was derivatized and injected during the same batch of the sample analysis. Measurement of 3HB was performed as previously described, on the 1260 system (Agilent) coupled to a 6500+ QTRAP (Sciex). MRM transition of the 3HB was Q1 (first quadruple mass filter) 238 *m/z* to Q3 238 *m/z* 23. Absolute concentrations (in μM) of 3HB were calculated from ion signal areas, with the calibration curve using a linear regression type. Accepted accuracies of calibration points were fixed between 80% and 120%, except for the lowest limit of quantification (LLOQ), which was set between 85% and 115%. In addition, the calibration curve was split into 2 ranges: low (from 46.49 μM to 464.92 μM) and high concentration (from 237.06 μM to 9335.7 μM). Each calibration curve (low and high) should have a least 4 points. Their ranges should cover calculated concentrations of biological samples, except basal ones. In this later case, concentrations were obtained with a ratio related to the lowest calibration point.

### Fecal DNA extraction and microbiota characterization.

Preparation and sequencing of fecal samples were performed at IHU Méditerranée Infection (Marseille, France). Briefly, DNA was extracted using 2 protocols. The first protocol consisted of physical and chemical lysis, using glass powder and proteinase K, respectively; then, processing was performed using the Macherey-Nagel DNA Tissue extraction kit (60).The second protocol was identical to the first protocol, with the addition of glycoprotein lysis and deglycosylation steps (61). The resulting DNA was sequenced, targeting the V3–V4 regions of the 16S rRNA gene as previously described (62). Raw FASTQ files were analyzed with Mothur pipeline v.1.39.5 for quality check and filtering (sequencing errors, chimeras) on a Workstation DELL T7910 (Round Rock). Raw reads (16,733,247 in total; on average, 120,383 per sample) were filtered (6,908,784 in total; on average, 49,703 per sample) and clustered into OTUs, followed by elimination of low-populated OTUs (until 10 reads) and by de novo OTU picking at 97% pair-wise identity using standardized parameters and SILVA rDNA Database v.1.19 for alignment. In all, 524 bacterial species were identified. Sample coverage was computed with Mothur and resulted to be, on average, higher than 99% for all samples; thus, it is a suitable normalization procedure for subsequent analyses. Bioinformatic and statistical analyses on recognized OTUs were performed with Python v.2.7.11. The most representative and abundant read within each OTU (as evidenced in the previous step with Mothur v.1.39.5) underwent a nucleotide Blast using the National Center for Biotechnology Information (NCBI) Blast software (ncbi-blast-2.3.0) and the latest NCBI 16S Microbial Database accessed (ftp://ftp.ncbi.nlm.nih.gov/blast/db/). A matrix of bacterial relative abundances was built at each taxon level (phylum, class, order, family, genus, species) for subsequent multivariate statistical analyses.

### Microbiota and OTU-level analyses.

Measurements of α diversity (within-sample diversity) such as observed_otus and Shannon index, were calculated at OTU level using the SciKit-learn package v.0.4.1. Exploratory analysis of β diversity (between-sample diversity) was calculated using the Bray-Curtis measure of dissimilarity calculated with Mothur and represented in PCoA, while — for hierarchical clustering analysis (HCA) — Bray-Curtis metrics and complete linkage method were implemented using custom scripts (Python v.2.7.11). In order to compare the microbiota taxa with gene expression datasets, a multivariate statistical Spearman correlation analysis (and related *P* values) was performed with custom Python scripts. Mann-Whitney *U* and Kruskall-Wallis tests were employed to assess significance for pair-wise or multiple comparisons, respectively, taking into account *P* ≤ 0.05 as significant. Correlations among relative abundance of candidate species and selected metabolites were estimated using Pearson correlation coefficient after a 2-stages Benjamini-Hochberg FDR of 10%. Analysis of similarity (ANOSIM, which represents the difference of datasets’ centroids) or, when indicated, Pearson correlation coefficient were computed with Python 2.7.11. LEfSe analysis was used to compare abundances of all bacterial species according to diet using the Kruskal-Wallis test (statistical significance was defined as *P* ≤ 0.05 after a 2-stages Benjamini-Hochberg FDR of 10%). Bacterial taxa with differential abundance between study groups were used as input for the LDA to calculate the effect size. LEfSe analysis at species level was performed with Mothur v.1.39.5 and graphed with Python 2.7.11.

### Flow cytometry on spleen and blood.

Spleens were harvested at the end of the experiment at early (day 12; D12) or late (D17) time point. Spleens were crushed in RPMI medium and subsequently filtered through a 100 μm cell strainer. In all cases, 2 million cells were preincubated with purified anti-mouse CD16/CD32 (clone 93, BioLegend, 16-0161-86) for 20 minutes at 4°C, before membrane staining. For intracellular staining, 4 million cells were used. Dead cells were excluded using the Zombie Aqua Fixable Viability Kit (BioLegend, 423102), and the eBioscience Foxp3/Transcription Factor Fixation/Permeabilization Concentrate and Diluent (Thermo Fisher Scientific, 00-5521-00) was used. Anti-mouse antibodies (clones, company, references) used for phenotyping T cells (TH1, Tc1, Treg) were: CD45 (30-F11, BioLegend, 103125), CD3e (145-2C11, BD Biosciences, 560527), CD4 (GK1.5, BioLegend, 100421), CD8a (53-6.7, BioLegend, 100761), CXCR3 (FAB1685P, Thermo Fisher Scientific, 17-1831-82), CD25 (PC61, BioLegend, 102038), CTLA-4 (UC10-4B9, Thermo Fisher Scientific, 12-1522-83), and FOXP3 (FJK-16s, Thermo Fisher Scientific, 56-5773-82). For phenotyping T cell exhaustion, the anti-mouse antibodies were CD45 (30F11, BioLegend, 109822), CD3 (17A2, BioLegend, 100222), CD4 (GK1.5, BioLegend, 100428), CD8a (53-6.7, BioLegend, 100722), Tim3 (B8.2C12B8.2C12), BioLegend, 134014), Lag 3 (C9B7W, BioLegend, 125210), CD137 (4-1 BB) (17B5, Thermo Fisher Scientific, 12-1371-83), and CD279 (PD-1) (J43, Thermo Fisher Scientific, 11-9985-85). For phenotyping DCs, the anti-mouse antibodies were CD45 (30F11, BioLegend, 109822), CD3 (145-2C11, BioLegend, 100326), CD4 (GK1.5, BioLegend, 100428), CD8a (53-6.7, BioLegend, 100761), CD11c (N418, BioLegend, 117318), F4/80 (BM8, BioLegend, 123117), CD11b (M1/70, BioLegend, 101257), PD-L1 (CD274) (MIH5, Thermo Fisher Scientific, 12-5982-82), and CD86 (GL1, BD Biosciences, 553691). Blood was harvested from mice 5 days after tumor and diet challenging. The blood was lysed by ACK and stained with CD45 (30-F11, BioLegend, 103128 ) and with negative selection with CD3 (17A2, BioLegend, 100222), CD19 (6D5, BioLegend, 115530), and NK (PK136, eBioscience, 108724). We selected monocytes with CD11b FITC (M1/70, Thermo Fisher Scientific, 11-0112-41) and CD11c (HL3, BD, 550283), and we observed the activation with CD69 (H1.2F3, BD Biosciences, 551113), CX3CR1 (SA011F11, BioLegend, 149033), CCR2 (SA203G11, BioLegend, 150605), and I-A /I-E (MS5/114.15.2, BioLegend, 107641). Samples were acquired on 13 color Cytoflex (Beckman Coulter), and analyses were performed with Kaluza software (Beckman Coulter).

### PD-L1 expression on RET cells by flow cytometry.

RET cells were grown and maintained in 6-well plates (200,000 cells/well) in the medium with conditions as described above. Cells were cultured with or without 10 ng/ mL of mouse rIFN-γ (Peprotech) and 10 mM 3HB (Ketoforce). After 48 hours, cells were harvested and stained with anti–PD-L1 (CD274) (MIH5, Thermo Fisher Scientific, 12-5982-82) Abs and istotype control Abs.

### GPR109 inhibition.

Mepenzolate bromide (C21H26BrNO3), the pharmacological inhibitor of GPR109A, was obtained from MilliporeSigma. Starting the day of RET challenge, the mepenzolate bromide was prepared and i.p. injected on a daily basis at 5 mg/mL (63) until mouse sacrifice.

### BM-DC and inducible immortalized DC.

The inducible immortalized DC (iniDC) line was a gift from Cornelia Richter (Technische Universitaet Dresden, Dresden, Germany). Basic medium for the culture of iniDC is RPMI 1640 medium, supplemented with FBS (10% v/v), penicillin (100 U/mL), streptomycin (100 μg/mL), 10 mM HEPES, 1 mM sodium pyruvate, and 50 μM β-mercaptoethanol (MilliporeSigma). Culture medium for the iniDCs was further supplied with 10 ng/mL GM-CSF (PeproTech, 315-03). As previously published by Richter et al. (64), iniDCs are immortalized under the induction of dexamethasone (Dex, MilliporeSigma, D0700000, final concentration at 100 nM) and doxycycline (Dox, MilliporeSigma, D3000000, final concentration at 2 μM). When Dex and Dox are removed from the medium, which is called deinduction, the iniDCs stop proliferation and enter into differentiation toward primary cell-like DCs, referred to as the de-iniDC. These cells were incubated with increasing dosages of 3HB (Ketoforce) ± 10 ng/mL of rIFN-γ (Peprotech) and stained with anti–PD-L1(CD274) Abs and isotype control Abs (MIH5, 12-5982-82). The same procedure was performed using BM-derived DC (BM-DC) cultivated from BM precursors using recombinant GM-CSF + IL-4 as previously reported (49).

### Statistics.

Data analyses and representations were performed either with the R software (65), Microsoft Excel (Microsoft Co.), or Prism 5 (GraphPad). For 2-group comparisons, Student’s *t* test (paired, 2 tailed) was utilized (Figure 1). For group comparisons of mice, statistical analyses gathering more than 2 groups were performed using Kruskall-Wallis with Dunn’s test to take into account multiple testing. For the log_10_-normalized data, we performed 2-way ANOVA with Sidak’s test (*P < 0.05); for 2-way ANOVA, we perform ANOVA followed with Dunnett’s test to take into account multiple testing. Otherwise, for 2 groups, statistical analyses were performed using the Mann-Whitney *U* test. Nonparametric tests were performed when the parametric assumptions did not hold (Kruskall-Wallis test with post hoc Dunn’s test, as well as Mann-Whitney *U* test). Outliers within a given distribution were tested using Grubbs’ test (66) with a threshold at *P* < 0.05. All tumor growth curves were analyzed using software developed in GK’s laboratory, and information about statistical analyses can be found in refs. 67 and 68. *P* values were 2 sided with 95% CI and were considered significant when *P* < 0.05. *P < 0.05, **P < 0.01, ***P < 0.001.

### Study approval.

All mice experiments were approved by the local institutional board (Board of Transgene) and performed in accordance with government and institutional guidelines and regulations (APAFIS# 21378-201907080848483459).

## Author contributions

GF conceived and designed the experiments. GF, MTA, PL, CT, CACS, CR, RD, and LZ performed the experiment and analyzed data. AGG, MF, and AF performed the cytometry experiment and analyzed cytometric data. SD, DL, and F. Aprahamian performed the metabolomics experiments. GF, CGI, and LD analyzed the metabolomics data. VI analyzed the metagenomics data and applied the statistical analysis. F. Asnicar and NS analyzed human data from PREDICT-1 and applied the statistical analysis. TS provided the human cohort PREDICT-1. OK analyzed data and improved the figures quality. BR provided materials for experiments. EC and MA conceived the human clinical cohort using KD. DD performed the statistical analyses. DR, via his metagenomic platform, performed the metagenomics experiments. GF, LD, GK, and LZ conceived the experiments and wrote the paper.

## Supplementary Material

Supplemental data

## Version 1 12/15/2020

In-Press Preview

## Version 2 01/25/2021

Electronic publication
