# Supplementary material for: Ketogenic diet and ketone bodies enhance the anticancer effects of PD-1 blockade
Source: JCI Insight. 2021 Jan 25;6(2):e145207. doi: 10.1172/jci.insight.145207 (PMC7934884; doi:10.1172/jci.insight.145207)
Supplement: Supplemental data [file jciinsight-6-145207-s237.pdf]

Ketogenic Diet and Ketone Bodies Enhance the Anticancer Effects of PD1 Blockade

Gladys Ferrere[1,], Maryam Tidjani Alou[1,] , Peng Liu[2], Anne-Gaëlle Goubet[1], Marine Fidelle[1], Oliver Kepp[2], Sylvère Durand[2], Valerio Iebba[3], Aurélie Fluckiger[1], Romain Daillère[4], Cassandra Thelemaque[1], Fanny Aprahamian[2], Déborah Lefevre[2,], Claudia Grajeda-Iglesias[1], Carolina Alves Costa Silva[1], Liwei Zhao[2], Bernhard Ryffel[5], Emeline Colomba[6], Monica Arnedos[6],Conrad Rauber[1], Didier Raoult[7], Francesco Asnicar[8], Tim Spector[9], Nicola Segata[8], Lisa Derosa[1], Guido Kroemer[2] and Laurence Zitvogel[1].

Supplemental materials

Figure S1: Metabolomics profiling of distant tissues (liver, heart).

Refer to Figure 2B, D. Heatmap of the non-supervised hierarchical clustering highlighting differences in the metabolic profiling of C57BL/6 mice fed normal diet (ND) versus ketogenic diet (KD) in heart (A) and liver (B) at day 12 post-start of KD versus ND. Each line represents one mouse metabolomics analysis.

Figure S2: Pharmacokinetics profiling of 3-hydroxybutyrate plasma concentrations during various diet interventions.

Longitudinal follow up of plasma concentrations of 3HB monitored in MALDI-TOF following various nutritional interventions in C57BL/6 mice [tumor-free (A) or RET tumor-bearers (B)] and in BALB/c mice depicted as area under the curve (AUC) (C). One to two experiments for each setting containing 6 mice/group has been performed. The diet was allowed in continuous (Cont) or with the intermittent On/off scheduling.

Figure S3: Metabolomics profiling of plasma when ketosis is blunted by the addition of sucrose.

Refer to Figure 3D-F. Heatmap of log2-fold changes in the non-supervised hierarchical clustering highlighting differences in the metabolic profiling of RET tumor-bearing C57BL/6 mice fed ketogenic diet (KD) +/- 10% sucrose in drinking water. Each line represents one mouse metabolomics analysis.

Figure S4: Ketogenic diet-induced changes in the intestinal taxonomic bacteria composition.

A. Volcano plot segregating significant over-or under-represented OTUs in normal diet (ND) versus ketogenic diet (KD) fed mice with p values and fold changes. B. Relative abundances of *A. muciniphila, R. lactatiformans, C. asparagiforme, P. capillosu, Turicibacter sanguinis and Lactobacilli* spp. in KD versus ND fed mice at day 12 represented as bar graphs of means +SEM in 6 mice/groups. C. Id. as in Figure 5A but showing the ND group.

Figure S5: Phenotyping of T cell splenocytes for inhibitory receptor expression .

A-B. Flow cytometry determination of various surface markers (PD-1, CTLA-4 ,4-1BB, Tim-3, Lag-3) expressed in CD4+ (A) and CD8+ (B) T cell subsets at day 15 in the spleens of BALB/c mice subjected to dietary interventions and cICB therapy. The results from 2 experiments comprising 6 mice/group are depicted, each dot representing one spleen. C. Blood monocyte enumeration. D. Phenotype gating in $CD11b^{+}CD11c^{-}/CD45^{+}$ in naive and RET tumor bearers at day 5 after starting ND, KD or 3HB per os. Each dot represents one mouse. One experiment out of 2-4 yielding similar results is presented. D. Flow cytometry determination of the cell surface expression of PD-L1 by RET cell line after a 48 hours exposure to 3HB +/-

rIFNg. A representative overlay of MFI is depicted, out of three leading to similar results. Statistics: Mann Whitney, Student's t-test. *$p<0.05$, **$p<0.01$, ***$p<0.001$.

Figure S6: Experimental settings for figure 8.

A-B. Orthotopic tumor model (RENCA-luciferase kidney cancer syngeneic of BALB/c (A)) and metastasis from lung cancer (TC-1-luciferase syngeneic of C57BL/6 mice (B)) were established 7 to 10 days prior to starting immunotherapy with anti-PD1 and/or anti-CTLA4 Abs. The diet interventions have been initiated either at the time of RENCA implantation or 9 days prior to iv injection of TC-1-luc. C. Assessment of memory responses in tumor free (cured) animals used from the experiments described in Figure 8 or naive animals as positive controls. Rechallenge with inoculation of 10 times the minimum tumorigenic dose of tumor cells.

A.Heart metabolites

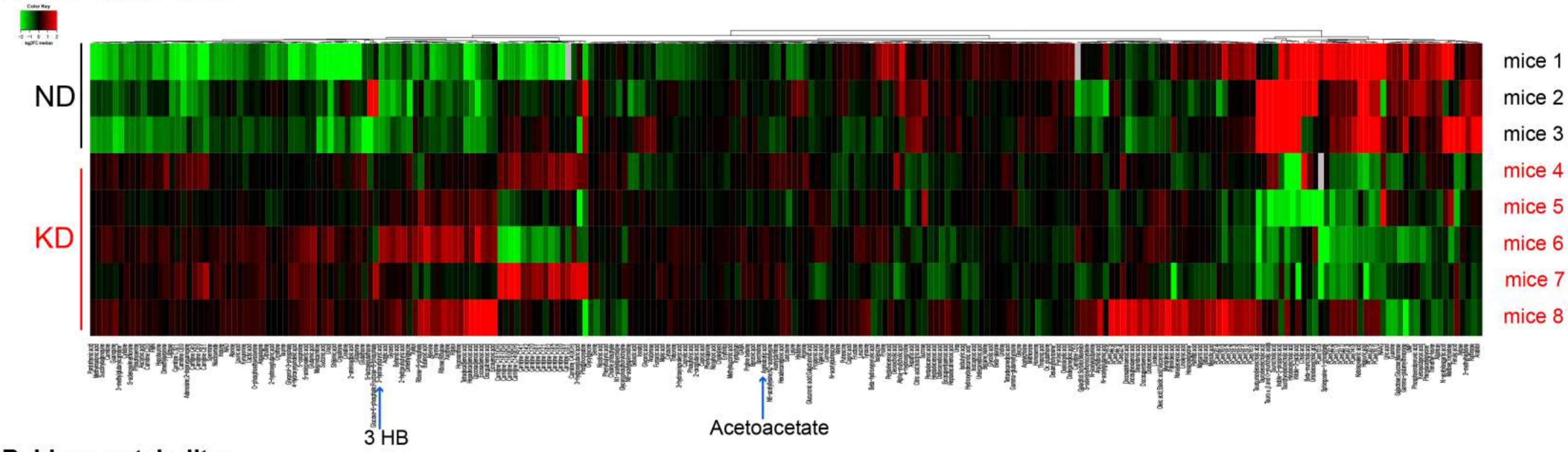

B. Liver metabolites

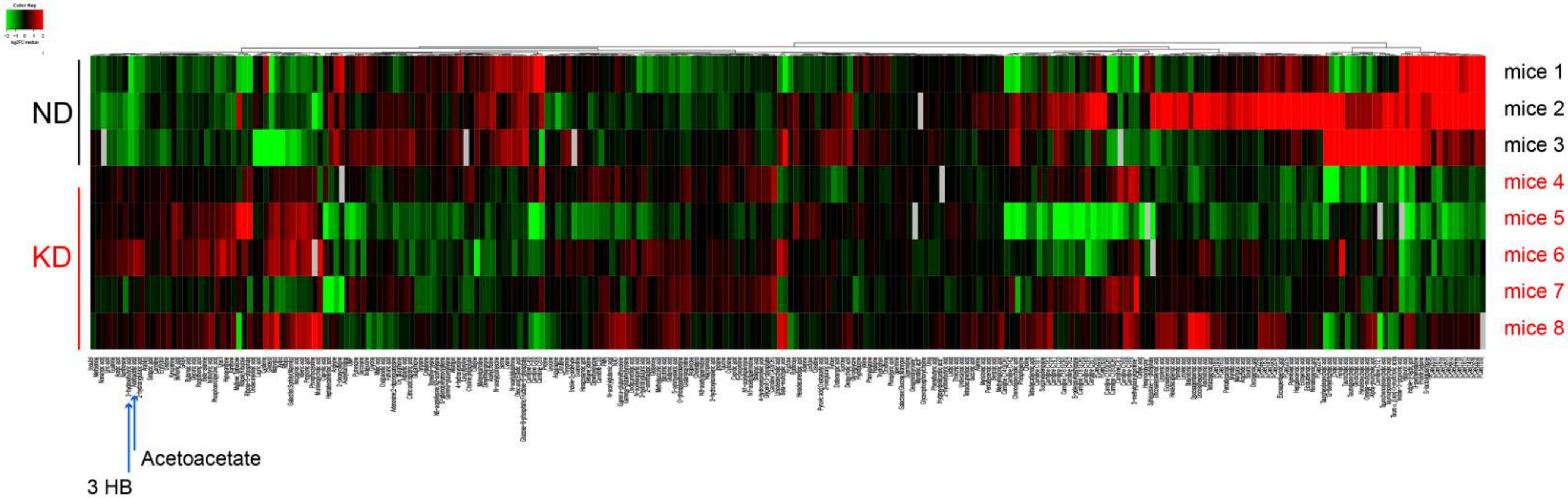

Figure S1: Metabolomics profiling of distant tissues (liver, heart).

Refer to Figure 2B, D. Heatmap of the non-supervised hierarchical clustering highlighting differences in the metabolic profiling of C57BL/6 mice fed normal diet (ND) versus ketogenic diet (KD) in heart (A) and liver (B) at day 12 post-start of KD versus ND. Each line represents one mouse metabolomics analysis.

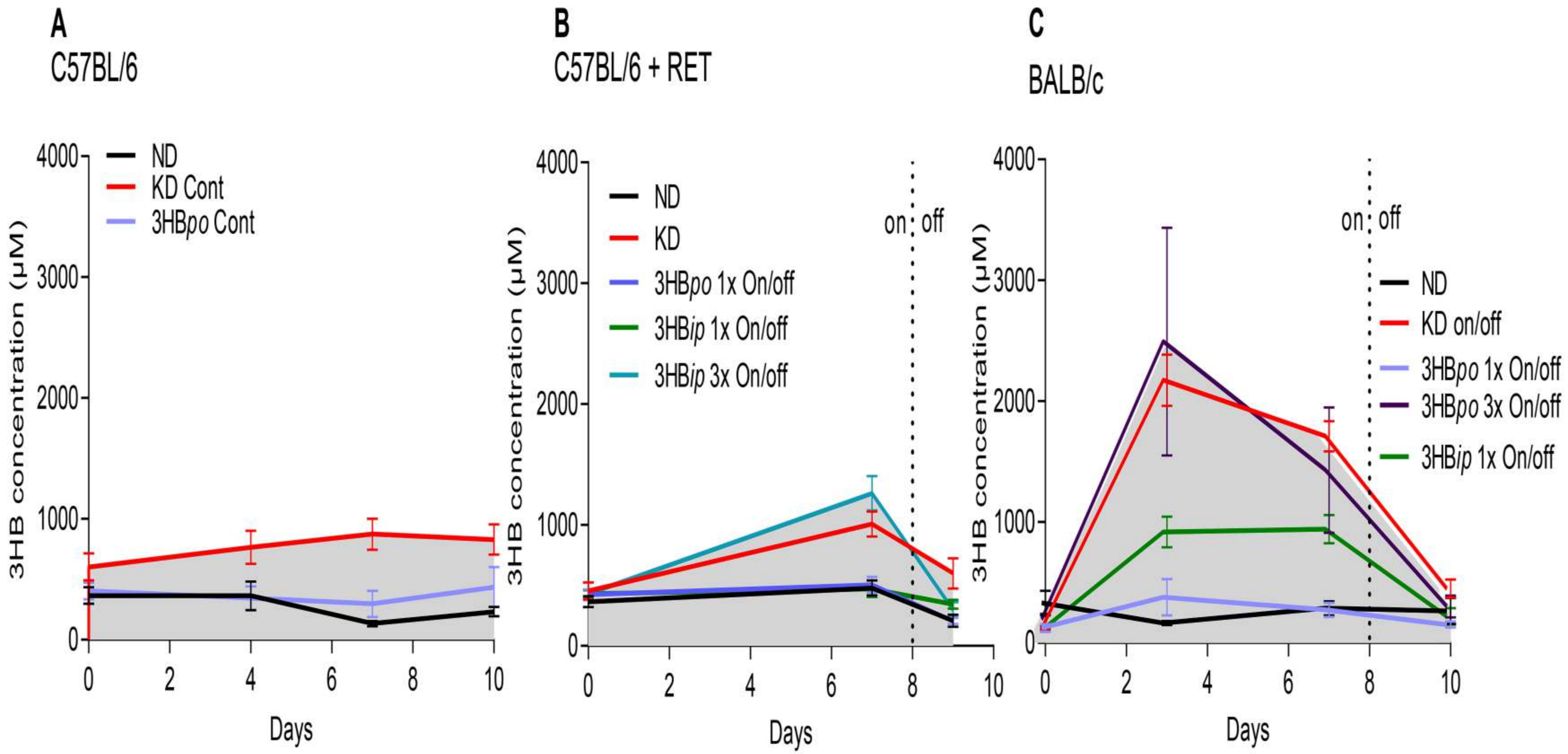

Figure S2: Pharmacokinetics profiling of 3-hydroxybutyate plasma concentrations during various diet interventions.

Longitudinal follow up of plasma concentrations of 3HB monitored in MALDI-TOF following various nutritional interventions in C57BL/6 mice [tumor-free (A) or RET tumor-bearers (B)] and in BALB/c mice depicted as area under the curve (AUC) (C). One to two experiments for each setting containing 6 mice/group has been performed. The diet was allowed in continuous (Cont) or with the intermittent On/off scheduling.

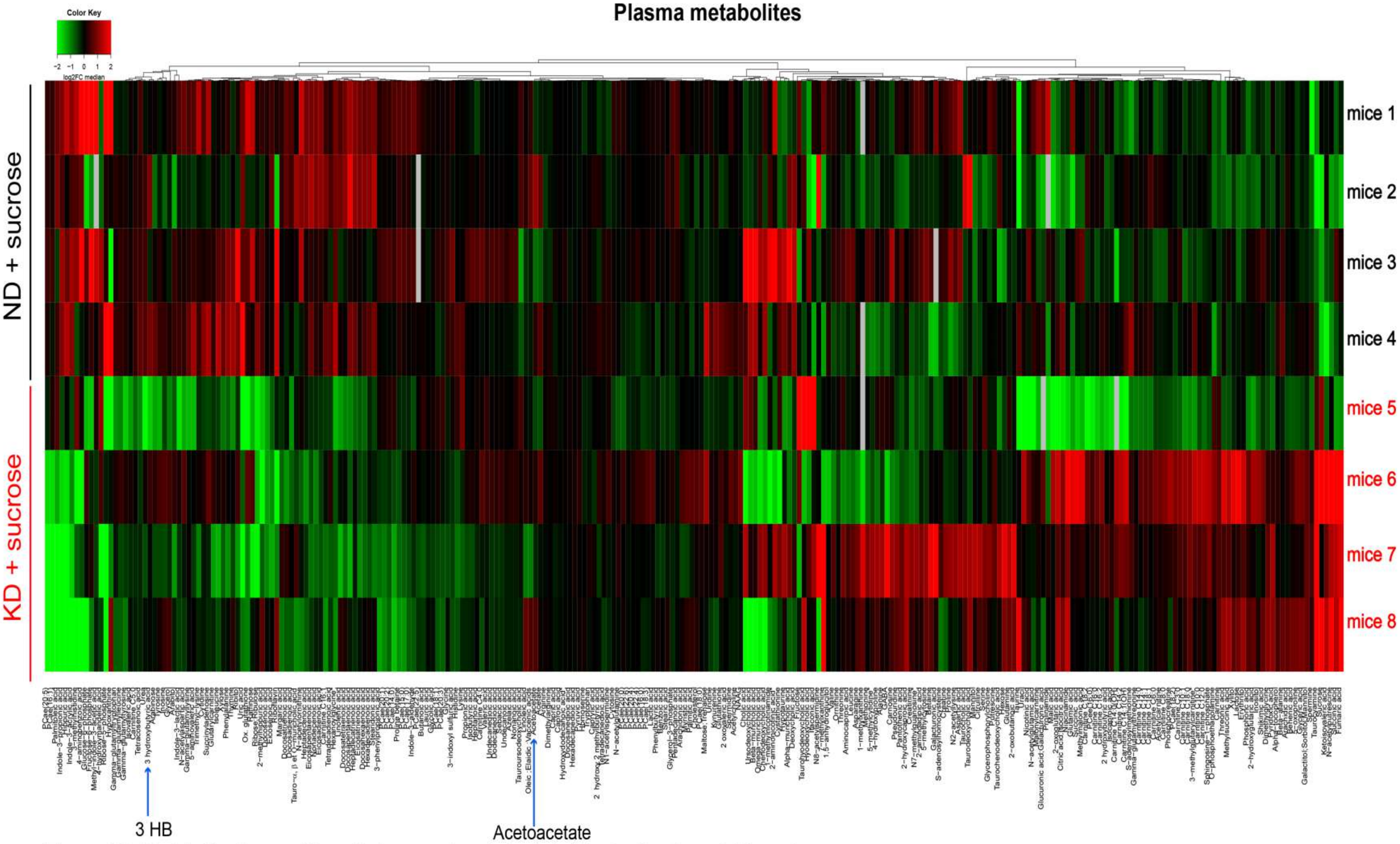

Figure S3: Metabolomics profiling of plasma when ketosis is blunted by the addition of sucrose.

Refer to Figure 3D-F. Heatmap of log2-fold changes in the non-supervised hierarchical clustering highlighting differences in the metabolic profiling of RET tumor-bearing C57BL/6 mice fed normal diet (ND) or ketogenic diet (KD) +/- 10% sucrose in drinking water. Each line represents one mouse metabolomics analysis.

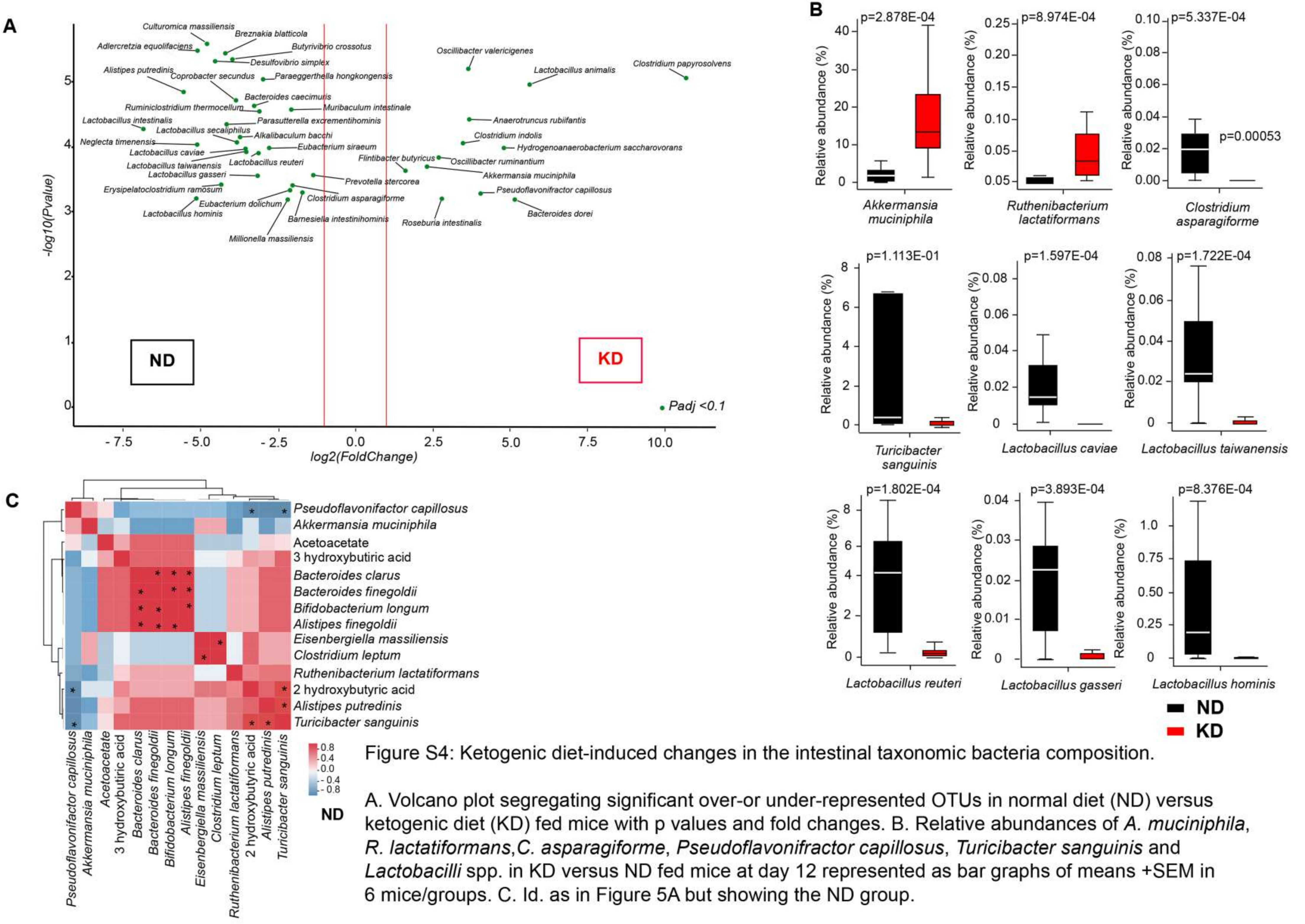

Figure S4: Ketogenic diet-induced changes in the intestinal taxonomic bacteria composition.

A. Volcano plot segregating significant over-or under-represented OTUs in normal diet (ND) versus ketogenic diet (KD) fed mice with p values and fold changes. B. Relative abundances of *A. muciniphila*, *R. lactatiformans*,*C. asparagiforme*, *Pseudoflavonifractor capillosus*, *Turicibacter sanguinis* and *Lactobacilli* spp. in KD versus ND fed mice at day 12 represented as bar graphs of means +SEM in 6 mice/groups. C. Id. as in Figure 5A but showing the ND group.

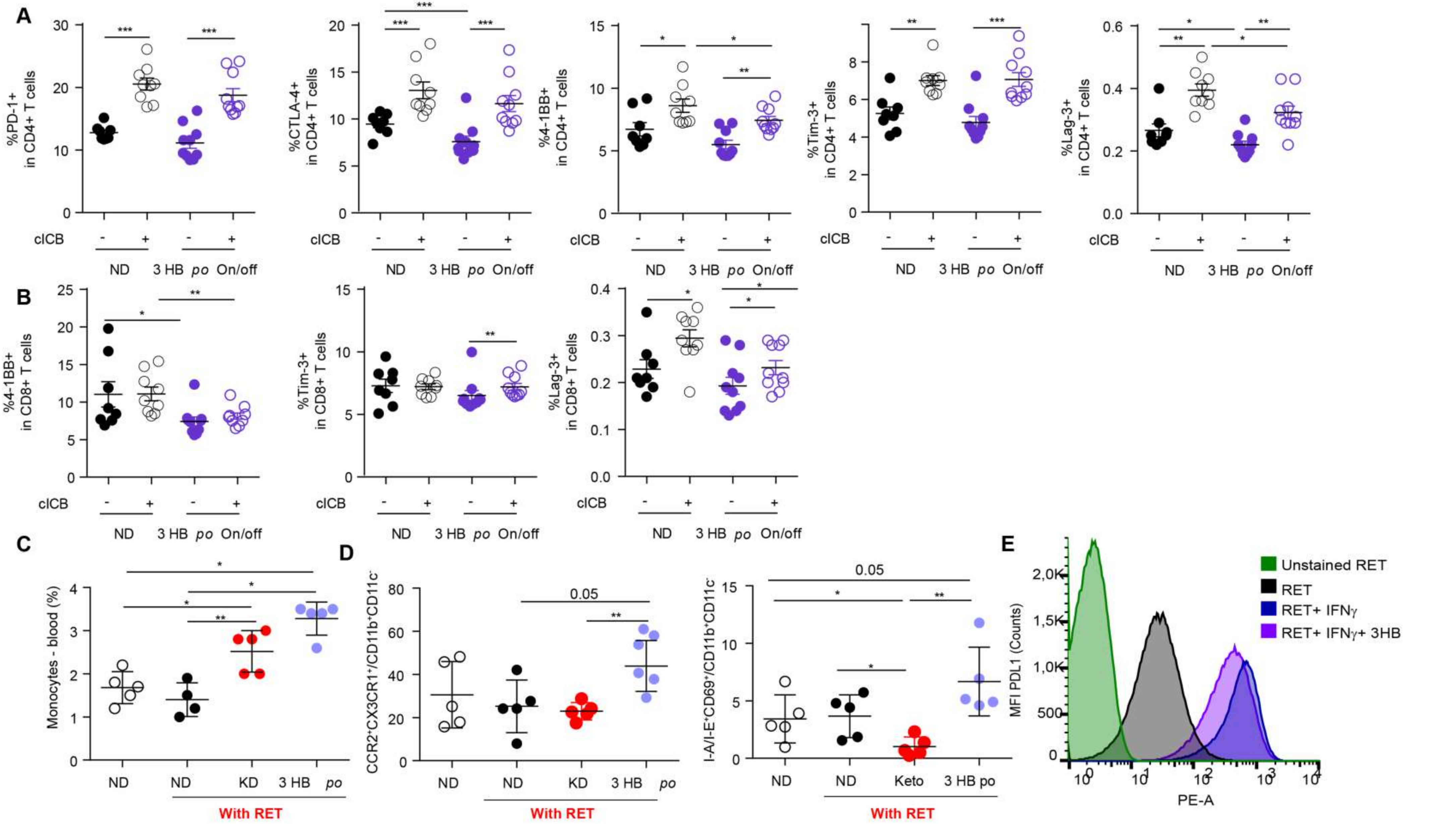

Figure S5: Phenotyping of T cell splenocytes for inhibitory receptor expression.

A-B. Flow cytometry determination of various surface markers (PD-1, CTLA-4 ,4-1BB, Tim-3, Lag-3) expressed in CD4+ (A) and CD8+ (B) T cell sub-sets at day 15 in the spleens of BALB/c mice subjected to dietary interventions and cICB therapy. The results from 2 experiments comprising 6 mice/-group are depicted, each dot representing one spleen. C. Blood monocyte enumeration.
D. Phenotype gating in CD11b+CD11c-/CD45+ in naive and RET tumor bearers at day 5 after starting ND, KD or 3HB per os. Each dot represents one mouse. One experiment out of 3 yielding similar results is presented. D. Flow cytometry determination of the cell surface expression of PD-L1 by RET cell line after a 48 hours exposure to 3HB +/- rIFNγ. A representative overlay of MFI is depicted, out of three leading to similar results.
Statistics: Mann Whitney, Student's t-test. *p<0.05, **p<0.01, ***p<0.001.

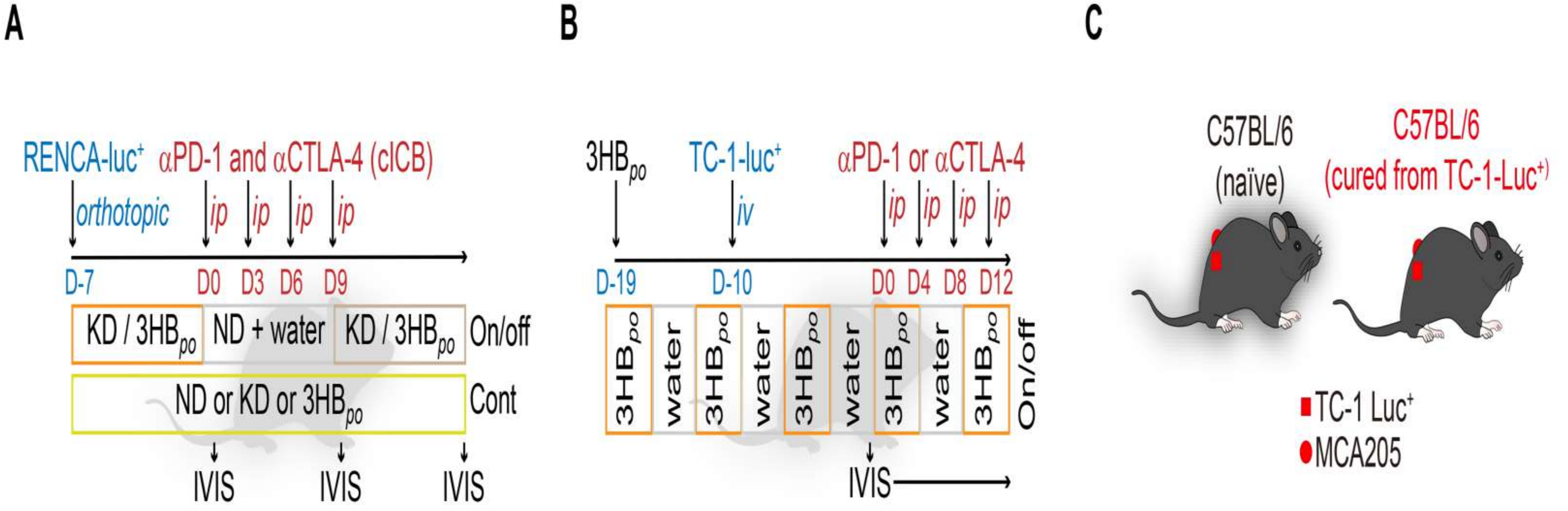

Figure S6: Experimental settings for figure 8.

A-B. Orthotopic tumor model (RENCA-luciferase kidney cancer syngeneic of BALB/c (A)) and metastasis from lung cancer (TC-1-luciferase syngeneic of C57BL/6 mice (B)) were established 7 to 10 days prior to starting immunotherapy with anti-PD1 and/or anti-CTLA4 Abs. The diet interventions have been initiated either at the time of RENCA implantation or 9 days prior to iv injection of TC-1-luc. C. Assessment of memory responses in tumor free (cured) animals used from the experiments described in Figure 8 or naive animals as positive controls. Rechallenge with inoculation of 10 times the minimum tumorigenic dose of tumor cells.

Supplemental table 1. List of metabolites differentially monitored in plasma of ketogenic diet-fed animals versus normal diet -fed littermates.

Cluster A	Cluster B					Cluster C
**Increased in ND**	**Increased in ND**	**Unchanged**			**Increased in KD**	**Increased in KD**
3.Methylhistidine ***	4.Hydroxyproline ***	2.aminoadipic.acid	Glycerol	Spermidine	Behenic.acid	2.hydroxybutyric.acid **
3.phenylpropionic.acid ***	Alanine *	2.hydroxyglutaric.acid	Glycerol.3.phosphate	Stearic.acid	Benzoic.acid	3.Methylglutarylcarnitine ***
5.aminovaleric.acid ***	Aminocaproic.acid *	2.oxoglutaric.acid	Glycylglycine	Tauro.alpha.muricholic/	Beta.hydroxypyruvic.acid	3.hydroxybutyric.acid ***
Acetylcholine ***	Creatinine *	2.hydroxy.3.methyl.butyric.acid	Hexacosanoic.acid	Tauro.beta.muricholic.acid/	Betaine.Valine	Acetate ***
Adenosine.2.deoxyguanosine **	Cytosine *	2.oxovaleric.acid	Hexadecadienoic.acid	Tauro.omega.muricholic.acid	Capric.acid	Acetoacetate ***
Allantoin ***	Deoxycholic.acid **	4.hydroxymandelic.acid	Hexadecanedioic.acid	Taurochenodeoxycholic.acid	Caprylic.acid	Acetylcarnitine ***
Alpha.muricholic.acid ***	Docosadienoic.acid ***	5.oxoproline	Homoserine	Taurocholic.acid	Decenoic.acid	Carnitine.C10.0 ***
Beta.muricholic.acid ***	Docosenoic.acid *	Alpha.tocopherol	Homovanillic.acid	Taurodeoxycholic.acid	Dodecenedioic.acid	Carnitine.C10.1 ***
Chenodeoxycholic.acid ***	Eicosenoic.acid **	Arabinose	Hydroxydodecanoic.acid	Tauroursodeoxycholic.acid	Glucose.6.phosphate.Fructose.6.phosphate	Carnitine.C12.0 ***
Cholic.acid ***	Erythritol *	Arabitol	Hydroxyphenyllactic.acid	Tetradecadienoic.acid	Glucuronic.acid.Galacturonic.acid	Carnitine.C14.0 ***
Creatine ***	GABA *	Arachidic.acid	Inosine	Tetradecanedioic.acid	Glutathione	Carnitine.C14.0.OH ***
Desaminotyrosine ***	Glycerophosphorylcholine **	Arachidonic.acid	Isobutyric.acid	Threonic.acid	Inositol	Carnitine.C14.1 ***
Docosahexaenoic.acid *	Glycine **	Arginine	Ketoisocaproic.acid	Thymine	Isocaproic.acid	Carnitine.C14.1.OH ***
Docosapentaenoic.acid ***	Heptadecatrienoic.acid *	Ascorbic.acid	Ketoisovaleric.acid	Trimethyl.lysine	Lauric.acid	Carnitine.C14.2 ***
Docosatetraenoic.acid ***	Heptadecenoic.acid ***	Asparagine	Kynurenic.acid	Tryptophan	N.acetylglutamic.acid	Carnitine.C16.0 ***
Docosatrienoic.acid ***	Histidine ***	Aspartic.acid	Kynurenine	Tyrosine	N.acetylglutamine	Carnitine.C16.1
Eicosadienoic.acid ***	Hypotaurine *	Azelaic.acid	Lactic.acid	UMP	N1.acetylspermidine	Carnitine.C16.2 ***
Eicosapentaenoic.acid ***	Indole.3.lactic.acid **	Beta.alanine	Linoleic.acid	Undecanedioic.acid	NAAG	Carnitine.C18.1 ***
Eicosatrienoic.acid ***	Isoleucine **	Butanoic.acid	Malic.acid	Undecanoic.acid	Orotic.acid	Carnitine.C18.2 **
Ferulic.acid ***	Leucine *	Caproic.acid	Margaric.acid	Uracil	Ox..glutathione	Carnitine.C4.0.OH ***
Hippuric.acid ***	Linolenic.acid *	Carnitine	Methylmalonic.acid	Uric.acid	Putrescine	Carnitine.C6.0 ***
Hyodeoxycholic.acid ***	Lysine ***	Carnitine.C3.0	Methylsuccinic.acid	Uridine	Ribose.Ribulose	Carnitine.C8.0 ***
Indole.3.acrylic.acid ***	Methionine ***	Carnitine.C4.0	Myristic.acid	Xylitol	Succinic.acid	Carnitine.C8.1 ***
Indole.3.aldehyde **	N6.acetyllysine.N2. acetyllysine ***	Carnitine.C5.0	Myristoleic.acid		Succinyladenosine	Citric.acid.Isocitric.acid **
Indole.3.propionic.acid ***	Nicotinic.acid **	Cholesterol	N.acetylaspartic.acid		Valeric.acid	Dimethylglycine ***
Omega.muricholic.acid ***	Nonadecenoic.acid ***	Citrulline	N.acetylputrescine			Fumaric.acid ***
Palmitoleic.acid ***	Oleic.acid.Elaidic.acid ***	Cytidine	N.glycolylneuraminic.acid			Hexanoylglycine
PCae.14.0 ***	PCae.16.0 ***	Dimethylarginine	Niacinamide			Hypoxanthine **
PCae.15.0 ***	PCae.18.0 **	Docosanedioic.acid	Nonanoic.acid			Lactose ***
PCae.16.1 ***	PCae.18.1 ***	Dodecanedioic.acid	O.phosphoethanolamine			Maltose.Trehalose **
PCae.17.0 ***	PCae.18.3 *	Dodecenoic.acid	Ornithine			Raffinose ***
PCae.20.0 ***	PCae.20.4 **	Fructose	Palmitic.acid			Ribose.5.phosphate ***
PCae.20.1 ***	Pentadecanoic.acid **	Galactitol.Sorbitol.Mannitol	Pantothenic.acid			Ribulose.5.phosphate ***
Cae.20.2 ***	Phenylalanine **	Galactose.Glucose.Mannose	PCae.18.2.			Sucrose ***
PCae.20.3 ***	Pyruvic.acid ***	Gamma.glutamylleucine	PCae.22.6.			Xanthine **
PCae.20.5 ***	Sphingosine.1.phosphate *	Gamma.glutamyllysine	Pentadecenoic.acid			2.hydroxybutyric.acid **
PCae.22.4 ***	Stearidonic.acid **	Gamma.glutamylthreonine	Phosphocreatine			3.Methylglutarylcarnitine ***
PCee.16.0 ***	Taurine *	Gamma.glutamyltryptophan	Phosphoric.acid			3.hydroxybutyric.acid ***
Proline.betaine ***	Threonine ***	Gamma.glutamyltyrosine	Proline			Acetate ***
Tetracosenoic.acid ***	Valine **	Glutamic.acid	Propionic.acid			Acetoacetate ***
Urea ***		Glutamine	Sebacic.acid			Acetylcarnitine ***
Ursodeoxycholic.acid ***		Glutarylcarnitine	Serine			Carnitine.C10.0 ***
Xylose ***		Glyceric.acid	Shikimic.acid			Carnitine.C10.1 ***

ND= Normal Diet KD = Ketogenic Diet

Table 1. List of metabolites differentially monitored in plasma of ketogenic diet-fed animals versus normal diet -fed littermates.
This table refers to the non-supervised hierarchical clustering of metabolites of C57BL/6 mice fed normal diet (ND) versus ketogenic diet (KD) in plasma (Data from Figure 2B) Metabolites increased in ND are in Cluster A (in green, left column), metabolites increased in KD are in Cluster C (in red, right column)Cluster B are metabolites with few modification or unchanged (in black, middle column).
